# Systematic Review of Childhood Sedentary Behavior Questionnaires: What do We Know and What is Next?

**DOI:** 10.1007/s40279-016-0610-1

**Published:** 2016-08-31

**Authors:** Lisan M. Hidding, Teatske M. Altenburg, Lidwine B. Mokkink, Caroline B. Terwee, Mai J. M. Chinapaw

**Affiliations:** 1 Department of Public and Occupational Health, EMGO Institute for Health and Care Research, VU University Medical Center, van der Boechorststraat 7, 1081BT Amsterdam, The Netherlands; 2 Department of Epidemiology and Biostatistics, EMGO Institute for Health and Care Research, VU University Medical Center, de Boelelaan 1089a, 1081HV Amsterdam, The Netherlands

## Abstract

**Background:**

Accurate measurement of child sedentary behavior is necessary for monitoring trends, examining health effects, and evaluating the effectiveness of interventions.

**Objectives:**

We therefore aimed to summarize studies examining the measurement properties of self-report or proxy-report sedentary behavior questionnaires for children and adolescents under the age of 18 years. Additionally, we provided an overview of the characteristics of the evaluated questionnaires.

**Methods:**

We performed systematic literature searches in the EMBASE, PubMed, and SPORTDiscus electronic databases. Studies had to report on at least one measurement property of a questionnaire assessing sedentary behavior. Questionnaire data were extracted using a standardized checklist, i.e. the Quality Assessment of Physical Activity Questionnaire (QAPAQ) checklist, and the methodological quality of the included studies was rated using a standardized tool, i.e. the COnsensus-based Standards for the selection of health Measurement INstruments (COSMIN) checklist.

**Results:**

Forty-six studies on 46 questionnaires met our inclusion criteria, of which 33 examined test–retest reliability, nine examined measurement error, two examined internal consistency, 22 examined construct validity, eight examined content validity, and two examined structural validity. The majority of the included studies were of fair or poor methodological quality. Of the studies with at least a fair methodological quality, six scored positive on test–retest reliability, and two scored positive on construct validity.

**Conclusion:**

None of the questionnaires included in this review were considered as both valid and reliable. High-quality studies on the most promising questionnaires are required, with more attention to the content validity of the questionnaires.

PROSPERO registration number: CRD42016035963.

**Electronic supplementary material:**

The online version of this article (doi:10.1007/s40279-016-0610-1) contains supplementary material, which is available to authorized users.

## Key Points

**Table:** 

In children and adolescents, no self-report or proxy-report sedentary behavior questionnaires are available that are both valid and reliable.
To improve the methodological quality of future studies, researchers need to adopt standardized tools such as COSMIN for the evaluation of measurement properties. In addition, reviewers and journal editors should also take into consideration whether such tools have been used when evaluating research articles.
Content validity needs more attention to ensure that questionnaires measure what they intend to measure.

## Introduction

Sedentary behavior is defined as activities performed in a seated or lying posture with very low energy expenditure (<1.5 metabolic equivalents [METs]) [1]. Sedentary behavior comprises a wide variety of activities, e.g. watching television, quiet play, passive transport, and studying. Excessive engagement in sedentary activities is seen in countries all over the world, i.e. 68 % of girls and 66 % of boys from 40 different countries in North America and Europe watch television for 2 or more hours per day [2]. Moreover, screen time seems to cover only a small part of the total time spent sedentary [3].

The relationship between sedentary behavior and health risks in children and adolescents is therefore of great interest. A recent review of reviews found strong evidence for an association between sedentary behavior and obesity in children [4]. Furthermore, moderate evidence for an association between blood pressure, physical fitness, total cholesterol, academic achievements, social behavioral problems, self-esteem, and sedentary behavior was found [4]. However, a major part of the existing evidence is based on cross-sectional studies, and subsequently no conclusion about causality can be drawn. Furthermore, sedentary behavior is often assessed using measurement instruments with inadequate or unknown measurement properties, and in some cases only screen time as an indicator of total sedentary time is assessed. Reviews examining the prospective relationship between sedentary behavior and different health outcomes concluded that there is no convincing evidence [5]. In addition, the evidence varied across type of measurement instrument and type of sedentary behavior [6].

Accelerometers and inclinometers are acknowledged as both valid and reliable instruments for measuring sedentary behavior in children and adolescents [7–9]; however, these measures are labor-intensive for researchers and are costly [10], and cannot provide information on the type and setting of sedentary behavior. Additionally, accelerometers cannot properly distinguish standing from sitting [11]. On the other hand, self- or proxy-report questionnaires are relatively inexpensive and easy to administer [10, 12]. Moreover, they can provide information on the type and setting of sedentary behavior. However, the use of questionnaires is not without limitations as social desirability and problems with accuracy of recall are factors of bias [12, 13].

A combination of objective measures, such as inclinometers providing information on duration and interruptions, and self-report providing information on the type and setting of sedentary behavior, would be optimal for measuring sedentary behavior. Different questionnaires for specific target populations have been developed, using different recall periods and formats, measuring different types and settings of sedentary behavior, and with different outcomes for measurement properties. This large variety of questionnaires available makes it difficult to choose the best instrument when conducting research; therefore, an overview of the measurement properties and characteristics of existing sedentary behavior questionnaires is highly warranted.

In 2011, Lubans et al. [7] reviewed studies examining the validity and reliability of questionnaires measuring sedentary behavior, indicating mixed results for both validity and reliability. As the amount of studies assessing the measurement properties of sedentary behavior questionnaires in children and adolescents has more than doubled since then, an update is required. Furthermore, an overview of the characteristics (e.g. target population, setting measured, recall period) of the included questionnaires was not incorporated in the review of Lubans et al., and studies in children under the age of 3 years were excluded [7]. Therefore, the aim of this review was to summarize studies that focused on assessing the measurement properties (e.g. validity, reliability, responsiveness) of self- or proxy-report questionnaires assessing (constructs of) sedentary behavior in children and adolescents under the age of 18 years, including a methodological quality assessment. Moreover, a summary of the questionnaire characteristics is provided.

## Methods

This review was registered at PROSPERO, the international prospective register of systematic reviews (registration number CRD42016035963), and the Preferred Reporting Items for Systematic Reviews and Meta-Analyses (PRISMA) reporting guidelines were followed.

### Literature Search

Systematic literature searches were carried out using the PubMed, SPORTDiscus (complete database up until December 2015), and EMBASE (complete database up until November 2015) databases. In PubMed, search terms were used in ‘AND’ combination and related to the following topics: ‘sedentary behavior’, ‘children’, (e.g. child, childhood, sedentary time, prolonged sitting), and ‘measurement properties’ (e.g. reliability, reproducibility, validity, responsiveness). The search was limited to humans and a variety of publication types (e.g. case reports, biography) were excluded (by using the ‘NOT’ combination). Free-text, Medical Subject Heading (MeSH), and Title/Abstract (TIAB) search terms were used. In SPORTDiscus, search terms regarding ‘children’ and ‘sedentary behavior’ were used in ‘AND’ combination. Search terms were used as title and abstract words. In EMBASE, both TIAB and EMTREE ‘sedentary behavior’ and ‘measurement properties’ search terms were used in ‘AND’ combination, and the EMBASE limits for children (e.g. infant, child) were applied (‘AND’ combination). In addition, reference lists and author databases were screened for additional studies. The full search strategies can be found in electronic supplementary material Appendix S1.

### Inclusion and Exclusion Criteria

Studies were included if they met the following criteria: (i) the study evaluated one or more of the measurement properties of a self- or proxy-report questionnaire, including sedentary behavior items; (ii) the aim of the questionnaire was to measure one or more of the constructs and dimensions of sedentary behavior; (iii) the average age of the study population was <18 years; and (iv) the study was published in the English language. Exclusion criteria were (i) studies examining questionnaires including physical activity and sedentary behavior items that had no separate score for sedentary behavior items; (ii) studies only reporting correlations between sedentary behavior constructs and non-sedentary constructs (e.g. correlation of self-reported or proxy-reported sedentary behavior with total activity counts measured by accelerometry); and (iii) studies evaluating the measurement properties of the questionnaire in a clinical sample.

### Selection Procedures

Two reviewers (TA and LH) independently selected studies of potential relevance based on titles and abstracts. Thereafter, both reviewers checked whether the full texts met the inclusion criteria. A third reviewer (MC) was consulted when inconsistencies arose.

### Data Extraction

Two independent reviewers (TA and LH) extracted data regarding the characteristics of the questionnaire under study, as well as the methods and results of the assessed measurement properties of the questionnaire, using structured forms. Disagreement between reviewers with respect to data extraction was discussed until consensus was reached.

Data regarding the questionnaire characteristics were extracted using the Quality Assessment of Physical Activity Questionnaire (QAPAQ) checklist, Part 1, which appraises the qualitative attributes of physical activity questionnaires [14]. Although originally developed for physical activity questionnaires, the QAPAQ checklist was also considered appropriate for sedentary behavior as physical activity and sedentary behavior questionnaires have similar structures and formats. Five of the nine checklist items were considered necessary to provide an informative summary of sedentary behavior questionnaires: (i) the constructs measured by the questionnaire, e.g. watching television, passive transport, quiet play, total sedentary behavior; (ii) the setting, e.g. at home, at school, leisure time; (iii) the recall period; (iv) the target population for whom the questionnaire was developed; and (v) the format, including the dimensions (i.e. duration, frequency), the number of questions, and the number and type of response categories. In addition, the following data regarding the methods and results of the assessed measurement properties were extracted: study sample, comparison measure, time interval, statistical methods, and results for each measurement property.

### Methodological Quality Assessment

Methodological quality of the studies was assessed using a slightly modified version of the COnsensus-based Standards for the selection of health Measurement INstruments (COSMIN) checklist with a 4-point scale (i.e. excellent, good, fair, or poor) [15–17]. Two independent reviewers (LH, and either MC, CT, or LM) assessed the methodological quality of each study, and disagreements were discussed until consensus was reached. The final methodological quality score was determined by applying the ‘worse score counts’ method (i.e. if one item was scored ‘poor’, the final score of the methodological quality was scored as ‘poor’) for each study separately.

Reliability, measurement error, internal consistency, and structural validity were rated using the designated COSMIN boxes, while convergent, criterion, and construct validity were rated as construct validity. None of the studies examined criterion validity, although this term was used in some studies that actually assessed construct validity. Content validity was not rated as too little information was available on the methods used for developing the questionnaire. Instead, a description of the questionnaire was included in the results section. None of the included studies examined the responsiveness of sedentary behavior questionnaires in children or adolescents.

One slight modification was applied to the original COSMIN, i.e. the percentage agreement was added as an excellent statistical method in the measurement error box as it is considered a parameter of measurement error rather than reliability [18]. For completing the reliability box, standards previously described by Chinapaw et al. [19] were used to assess the appropriateness of the time interval in a test–retest reliability study; i.e. (i) questionnaires recalling a usual week should have a time interval between >1 day and <3 months; (ii) questionnaires recalling the previous week should have a time interval between >1 day and <2 weeks; and (iii) questionnaires recalling the previous day should have a time interval between >1 day and <1 week.

### Questionnaire Quality Assessment

#### Reliability

Reliability refers to the extent to which scores for persons who have not changed are the same, with repeated measurement under several conditions [20]. The outcomes regarding reliability of the included questionnaires were seen as acceptable in the following situations: (i) an outcome of >0.70 for intraclass correlations and kappa values [21]; or (ii) an outcome of >0.80 for Pearson and Spearman correlations as a result of not taking systematic errors into account [22]. For an adequate measurement error the smallest detectable change (SDC) should be smaller than the minimal important change (MIC) [21]. Internal consistency was considered acceptable when Cronbach’s alphas were calculated on unidimensional scales and were between 0.70 and 0.95 [21].

The majority of studies provided separate correlations for the different constructs of sedentary behavior, as presented in the questionnaire, e.g. providing separate correlations for watching television, passive transport, and reading. Therefore, to obtain a final reliability rating, an overall evidence rating was applied in the present review, incorporating all available correlations for each questionnaire per study. A questionnaire received a positive evidence rating (+) when there were ≥80 % acceptable correlations, a mixed evidence rating (+/−) when the acceptable correlations were ≥50 and <80 %, and a negative rating (−) when there were <50 % acceptable correlations. No evidence rating for measurement error could be conducted as information on the MIC is currently lacking for all included questionnaires, which is needed for interpretation of the findings. Therefore, only a description of results is given.

#### Validity

Validity refers to the degree to which a measurement instrument measures what it is supposed to measure [20]. Validity concerns three measurement properties, i.e. content validity, structural validity, and construct validity. Content validity refers to the degree to which the content of a questionnaire adequately reflects the constructs to be measured [20]; structural validity refers to the degree to which the scores of a questionnaire are an adequate reflection of the dimensionality of the construct to be measured [20]; and construct validity refers to the degree to which the scores of a measurement instrument agree with hypotheses, e.g. agreement with scores of another measurement instrument [20]. In case of structural validity, a factor analysis was considered appropriate if the explained amount of variance by the extracted factors was at least 50 % of when the comparative fit index (CFI) was >0.95 [21, 22]. However, as most of the included construct validity studies lacked a priori formulated hypotheses it was unclear what was expected, making it difficult to interpret these results. Table 1 presents the criteria for judging the results of construct validity studies. Level 1 indicates strong evidence, level 2 indicates moderate evidence, and level 3 indicates weak evidence, yet worthwhile to investigate further. Similar to the reliability rating, an overall evidence rating for construct validity was applied, incorporating all available correlations provided for each questionnaire per study. As no hypotheses for validity were available in relation to mean differences and limits of agreement, only a description of the results is included in Sect. 3.

**Table 1:** Constructs of sedentary behavior measured by the questionnaires evaluating construct validity, subcategorized by level of evidence and criteria for acceptable correlations

Constructs of sedentary behavior measured	Level 1	Level 2	Level 3
Sedentary behavior, all constructs (i.e. including at least screen time and non-screen leisure time activities, e.g. quiet play/hobbies/social activities, school/study time, and passive transport)	ActivPAL ≥0.70 Direct observation ≥0.70	Accelerometer 100 cpm ≥0.60	Questionnaire, diary, interview: corresponding constructs ≥0.70 Accelerometer lower or higher than 100 cpm ≥0.40
Sitting (overall time)	ActivPAL ≥0.70 Direct observation ≥0.70	Accelerometer 100 cpm ≥0.50	Questionnaire, diary, interview: corresponding constructs ≥0.70 Accelerometer lower or higher than 100 cpm ≥0.40
TV watching time/screen time	Direct observation ≥0.70	Diary, logs ≥0.60 TV monitoring device ≥0.60	Questionnaire, interview: corresponding constructs ≥0.70 Accelerometer ≥0.40
Sedentary behavior, not all constructs or time frames (e.g. excluding screen time or time spent at school)	Direct observation ≥0.70 ActivPAL ≥0.70	Accelerometer 100 cpm ≥0.60^a^ Accelerometer 100 cpm ≥0.50^b^	Questionnaire, diary, interview: corresponding constructs ≥0.70 Accelerometer lower or higher than 100 cpm ≥0.40

^a^Time frame of questionnaire matches that of the accelerometer (e.g. both measures included total daytime)

^b^Time frame of questionnaire (e.g. data included parts of daytime or excluded classroom sitting) does not match that of the accelerometer (e.g. data included total daytime, or all sedentary constructs)

## Results

**Fig. 1:**
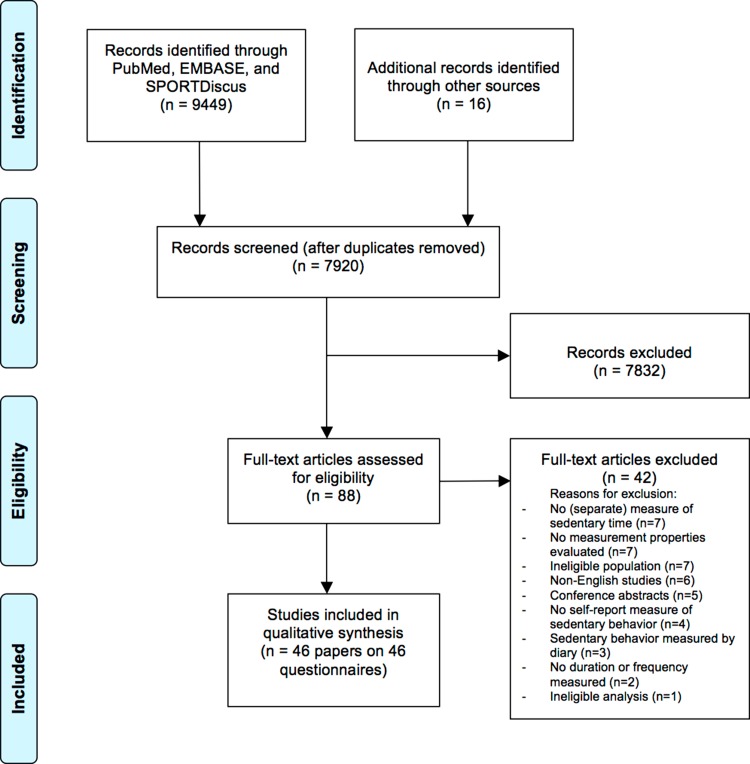
PRISMA flow diagram of study inclusion process. *PRISMA* preferred reporting items for systematic reviews and meta-analyses

A total of 3049, 4384, and 2016 studies were identified in the PubMed, EMBASE, and SPORTDiscus databases, respectively. After removing duplicates, 7904 studies remained. After screening titles and abstracts, 72 full-text papers were assessed for eligibility, of which 30 met the inclusion criteria. Another 16 studies were found through cross-reference searches. Eventually, 46 studies on 46 questionnaires were included (Fig. 1), of which 33 assessed test–retest reliability, nine assessed measurement error, two assessed internal consistency, 22 assessed construct validity, eight assessed content validity, and two assessed structural validity. Two of the included questionnaires were assessed by two studies, i.e. the Patient-Reported Outcome Measurement Information System [23, 24] and the Girls Health Enrichment Multi-site Studies Activity Questionnaire [25, 26]. In addition, multiple modified versions of questionnaires were examined by the included studies, i.e. two versions of the Canadian Health Measures Survey [27, 28], the Adolescent Sedentary Activity Questionnaire [29, 30], the International Physical Activity Questionnaire–Short Form [31, 32], and the Youth Risk Behavior Survey [34, 35]. Furthermore, three versions of the Self-Administered Physical Activity Checklist [36–38] and the Health Behavior in School-aged Children were included [39–41]. The remaining questionnaires were only examined by one single study.

### Description of Questionnaires

Electronic supplementary material Table S1 provides a description of the included questionnaires, stratified by age group, i.e. preschoolers younger than 6 years of age, children aged between 6 and 12 years, and adolescents from the age of 12 years. Of the included questionnaires, 8 were designed for preschoolers, 24 were designed for children, and 14 were designed for adolescents. Nineteen of the questionnaires merely focused on screen time, while 27 focused on a variety of constructs of sedentary behavior. Response categories were mostly categorical (e.g. Likert scale) or continuous (e.g. time spent, in hours and/or minutes). Recall periods varied across questionnaires, including past few months, last week, previous day, and a usual/habitual/typical day/week.

### Test–Retest Reliability

Table 2 summarizes the test–retest reliability studies, of which four were in preschoolers, 18 were in children, and 11 were in adolescents and older children. None of the studies received an excellent methodological quality rating, 9 had a good rating, 17 had a fair rating, 6 had a poor rating, and 1 of the studies received both a fair rating and a poor rating due to the use of multiple time intervals. A small sample size and no description of how missing items were handled were the major reasons for the low methodological quality ratings. In preschoolers, the Energy Balance-Related Behaviors self-administered primary caregiver questionnaire [42] seemed the most reliable, currently available questionnaire for assessing sedentary behavior, although the methodological quality of this study was only rated as fair and the evidence was mixed. For children and adolescents, the most reliable, currently available questionnaires were the Sedentary Behavior and Sleep Scale [43] (i.e. good methodological quality, mixed evidence rating) and the Adolescent Sedentary Activity Questionnaire (Brazilian version) [30] (i.e. fair methodological quality, positive evidence rating), respectively.

**Table 2:** Test–retest reliability of sedentary behavior questionnaires for youth sorted by age category, methodological quality, and evidence rating

Questionnaire	Study population^a^	Time interval	Results	Methodological quality^b^	Evidence rating
Preschoolers (mean age <6 years)					
Preschool-aged Children’s Physical Activity Questionnaire (Pre-PAQ) [proxy] [48]	*n* = 103 Age: 3- to 5-year-olds Sex: 48 % girls	1–2 weeks	Family car behavior: ICC 0.31–0.63	Good	−
Energy balance-related behaviors (ERBs) self-administered primary caregivers questionnaire (PCQ), from the ToyBox study (proxy) [42]	*n* = 93 preschoolers	2 weeks	Computer use: ICC: weekdays 0.716, weekend days 0.812 Active/passive transport: ICC: travel forth 0.913, time 0.820, travel home 0.882, time 0.892 TV viewing: ICC: weekdays 0.674, weekend days 0.667 Quiet play: ICC: weekdays 0.421, weekend days 0.501	Fair	+/−
KidActive-Q (proxy) [59]	*n* = 20 Age: 4.2 ± 1.3 years (2–6) Sex: 50 % girls	3 weeks	Watching TV: ICC 0.85 (95 % CI 0.72–0.97)	Poor	+
Physical activity questionnaire for parents of preschoolers (translated from Spanish) [49]	*n* = 21 Age: 3- to 5-year-olds Sex: percentage of girls unknown	1 week	Duration low activity: *r* = 0.86	Poor	+
Children (mean age ≥6 and <12 years)					
Sedentary Behavior and Sleep Scale (SBSS) [proxy] [43]	*n* = ranging from 59 to 62 Age: 6.7 ± 0.5 years (total sample) Sex: 59 % girls (total sample)	1 week	Homework: ICC: weekday 0.62, weekend 0.79 Tuition: ICC: weekday 0.68, weekend 0.73 Screen time: ICC: weekday 0.70, weekend 0.59 Total sedentary time: ICC: weekday 0.72, weekend 0.61	Good	+/−
A TV viewing question (proxy) [60]	*n* = 67 Age: 4- to 7-year-olds Sex: percentage of girls unknown	2–8 weeks	TV viewing: Kappa 0.53 (95 % CI 0.35–0.74); SCC 0.68; ICC 0.70 (95 % CI 0.55–0.80)	Good	−
Children’s Leisure Activities Study Survey–Chinese version questionnaire (CLASS–C) [54]	*n* = 214 Age: 10.9 ± 0.9 years (9–12) Sex: 62 % girls	1 week	Weekly sedentary time [min]: ICC 0.69 (95 % CI 0.59–0.77)	Good	−
TV viewing questionnaire (proxy) [61]	*n* = 133 Age: 5- to 6-year-olds and 10- to 12-year-olds Sex: percentage of girls unknown	7–14 days	TV viewing: ICC 0.78 (95 % CI 0.69–0.84) [dichotomized into ≤2 h/day and >2 h/day]	Fair	+
Energy Retention Behavior Scale for Children (ERB–C scale) [46]	*n* = 36 Age: same approximate age as the validity study: 9.6 ± 0.6 years Sex: 56 % girls	Twice-weekly intervals	Sedentary-behavior scale: ICC 0.81	Fair	+
The Adolescents Sedentary Activities Questionnaire (ASAQ) [29]	*n* = 250; Age: 11.3 (*n* = 98), 13.3 (*n* = 73), and 15.3 years (*n* = 79) Sex: 49, 47, and 37 % girls, respectively	2 weeks	Total week: ICC: grade 6 boys 0.57 (95 % CI 0.25–0.76), girls 0.86 (95 % CI 0.75–0.92); grade 8 boys 0.84 (95 % CI 0.69–0.91), girls 0.70 (95 % CI 0.40–0.85); grade 10 boys 0.72 (95 % CI 0.52–0.84), girls 0.82 (95 % CI 0.63–0.92)	Fair	+
TV viewing items of the Health Behavior in School-Aged Children survey (HBSC) [39]	*n* = 112 Age: 11.8 ± 0.6 years Sex: 48 % girls	1 week	Mean TV viewing time: ICC: boys 0.76 (95 % CI 0.63–0.85); girls 0.81 (95 % CI 0.69–0.88)	Fair	+
Selected indicators from the Health Behavior in School-aged Children (HBSC) questionnaire [40]	*n* = 95 Age: 11.7 ± 0.4 years (*n* = 44)/15.8 ± 0.3 years (*n* = 51) Sex: 55 % girls (*n* = 44) 39 % girls (*n* = 51)	3 weeks	Watching TV: ICC: school days 0.72 (95 % CI 0.61–0.81), weekends 0.74 (95 % CI 0.63–0.83) Doing homework: ICC: school days 0.78 (95 % CI 0.68–0.85), weekends 0.73 (95 % CI 0.62–0.82) Playing PC or console games: ICC: school days 0.54 (95 % CI 0.38–0.67), weekends 0.69 (95 % CI 0.57–0.78) Using the PC: ICC: school days 0.33 (95 % CI 0.14–0.50), weekends 0.50 (95 % CI 0.33–0.64)^c^	Fair	+/−
UP4FUN child questionnaire [62]	*n* = 143 Age: 10- to 12-year-olds Sex: 57 % girls	1 week	TV/DVD watching: ICC: weekdays 0.77, weekend days 0.74, yesterday 0.66 Computer/game console: ICC: weekdays 0.84, weekend days 0.80, yesterday 0.67 Breaking up TV/DVD watching: ICC 0.72 Breaking up computer/game console: ICC 0.70 Breaking up school lesson: ICC 0.68	Fair	+/−
Selected physical activity and sedentary behavior items of the Health Behavior in School-aged Children (HBSC) questionnaire [41]	*n* = 693 Age: 11.08 ± 0.45 years/15.12 ± 0.45 years Sex: 49 % girls	Czech Republic and Slovakia: 4 weeks Poland: 1 week	4-week interval: ICC: TV use—weekday 0.51, weekend day 0.52; computer use—weekdays 0.61, weekend days 0.62; sitting time—weekdays 0.55, weekend days 0.53 4-week interval: Cohen’s kappa: TV use—weekday 0.45, weekend day 0.41; computer use—weekdays 0.49, weekend days 0.51; sitting time—weekdays 0.42, weekend days 0.39 1-week interval: ICC: TV use—weekday 0.66, weekend day 0.88; computer use—weekdays 0.80, weekend days 0.88; sitting time—weekdays 0.91, weekend days 0.92	Fair	−
Self-Administered Physical Activity Checklist (SAPAC) (modified) [37]	*n* = 103 Age: 11.7 ± 0.5 years Sex: 50 % girls	Minimum of 5 days	Boys: ICC: TV/video 0.20, PC 0.40, total 0.36 Girls: ICC: TV/video 0.38, PC 0.35, total 0.34	Fair	−
The Eating and Activity Questionnaire Trial (Project EAST) and a modified question of the Youth Risk Behavior Survey (YRBS)^d^ [51]	*n* = 245 Age: 11- to 15-year olds Sex: 41 % girls	1 week	Weekday TV, school year:^e^ Exact agreement—kappa 0.55; +1 category—kappa 0.59; percentage of children meeting recommendation of <2 h/day TV viewing: SCC 0.68 Weekend TV, school year: Exact agreement—kappa 0.51; +1 category—kappa 0.40; percentage of children meeting recommendation of <2 h/day TV viewing: SCC 0.61 Weekday TV, summer: Exact agreement—kappa 0.46; +1 category—kappa 0.39; percentage of children meeting recommendation of <2 h/day TV viewing: SCC 0.58 Weekend TV, summer: Exact agreement—kappa 0.42; +1 category—kappa 0.40; percentage of children meeting recommendation of <2 h/day TV viewing: SCC 0.55 Computer use: Exact agreement—kappa 0.49; +1 category—Kappa 0.56; percentage of children meeting recommendation of <2 h/day TV viewing: SCC 0.60	Fair	−
Girls health Enrichment Multi-site Studies (GEMS) Activity Questionnaire (GAQ) [25]	*n* = 68 Age: 9.0 ± 0.6 year Sex: 100 % girls	4 days	TV watching: PCC—yesterday 0.3454, usual 0.3827 Other sedentary activities: PCC—yesterday 0.469, usual 0.4837	Fair	−
The ENERGY-project Dietary and Physical Activity Habits of Children–child questionnaire [53]	*n* = 730 Age: (11.3 ± 0.5–12.6 ± 0.6 years) Sex: 47–58 % girls	1 week	TV watching: ICC: weekdays 0.67, weekend days 0.68, yesterday 0.68 Computer use: ICC: weekdays 0.67, weekend days 0.67, yesterday 0.54 Travel by car to school: ICC 0.91 Transport today to school: ICC 0.79 Travel by public transport to school: ICC 0.88 Activity during breaks: ICC 0.80	Fair	−
Parent proxy-report of physical activity and sedentary activities (proxy) [63]	*n* = 147 Age: 6- to 10-year-olds; 13- to 14-year olds Sex: 51 % girls (in total sample *n* = 189)	2 and 6 months	2-month interval: ICC: watching TV 0.60 (95 % CI 0.49–0.79); sitting at a computer 0.61 (95 % CI 0.50–0.71); doing homework 0.56 (95 % CI 0.45–0.67); reading 0.64 (95 % CI 0.54–0.73); playing a musical instrument 0.34 (95 % CI 0.20–0.49); playing quietly 0.42 (95 % CI 0.29–0.55); traveling by car/public transport 0.49 (95 % CI 0.36–0.62) 6-month interval: ICC: watching TV 0.49 (95 % CI 0.36–0.62); sitting at a computer 0.44 (95 % CI 0.30–0.57); doing homework 0.59 (95 % CI 0.48–0.70); reading 0.54 (95 % CI 0.42–0.66); playing a musical instrument 0.59 (95 % CI 0.48–0.71); playing quietly 0.32 (95 % CI 0.17–0.47); traveling by car/public transport 0.43 (95 % CI 0.29–0.57)	2-month interval: Fair 6-month interval: Poor	−
Quantification de l’Activite Physique en Altitude Chez le Enfants (QAPACE) [64]	*n* = 121 Age: 9.1 ± 0.8 years (8–10); 12.1 ± 0.8 years (11–13); 15.0 ± 0.8 years (14–16) Sex: 54 % girls	90 days	Classroom sitting: ICC 0.97 (95 % CI 0.96–0.98)	Poor	+
Girls health Enrichment Multi-site Studies (GEMS) Activity Questionnaire (GAQ) [26]	*n* = 172 Age: 8.8 ± 0.8 years Sex: 100 % girls	110.3 ± 17.6 days (average no. of days ± SD)	TV watching: PCC: yesterday 0.13, usual 0.31 Other sedentary activities: PCC: yesterday 0.32, usual 0.30	Poor	−
Sedentary behavior items from a new questionnaire to identify usual patterns of physical activity (proxy and non-proxy) [33]	*n* = 69 Age: 9.9 years (8.5–12.7) Sex: 100 % girls *n* = 47 mothers *n* = 35 fathers	Girls: 12–16 days Parents: 12–28 days	Sitting, school day: ICC: girls 0.35, mothers 0.21, fathers 0.20 Sitting, weekend day: ICC: girls 0.36, mothers 0.25, fathers 0.24 TV, school day: ICC: girls 0.84, mothers 0.45, fathers 0.86 TV, weekend day: ICC: girls 0.81, mothers 0.82, fathers 0.79	Poor	−
Older children and adolescents (mean age ≥12 years)					
School Health Action Planning and Evaluation System (SHAPES) physical activity questionnaire [50]	*n* = 1636 Age: 9- to 12-graders Sex: 55 % girls	1 week	Sedentary activities item domain: Kappa 0.55 ± 0.01 Weekly screen-time: Kappa 0.51	Good	−
Activity Questionnaire for Adults and Adolescents (AQuAA) [65]	*n* = 53 Age: 14.1 ± 1.4 years Sex: 43 % girls	2 weeks	Sedentary activities: ICC 0.57 (95 % CI 0.34–0.73)	Good	−
Child and Adolescent Physical Activity and Nutrition Survey (CAPANS-PA) recall questionnaire [66]	*n* = 77 Age: 12 ± 0.8 years (11–14) Sex: 51 % girls	7 days	All sedentary activities: ICC: Monday–Friday 0.43 (95 % CI 0.21–0.61), Saturday 0.57 (95 % CI 0.38–0.71), Sunday 0.65 (95 % CI 0.48–0.77)	Good	−
International Physical Activity Questionnaire–Short Form (IPAQ-SF) [Chinese version] [32]	*n* = 92 Age: 15.9 ± 1.4 years Sex: 53 % girls	1 week	Sitting: ICC: All 0.32 (95 % CI 0.12–0.49), boys 0.06 (95 % CI −0.24 to 0.35), girls 0.43 (95 % CI 0.17–0.63)	Good	−
1999 Youth Risk Behavior Survey (YRBS) questionnaire [34]	*n* = 4619 Age: 13- to 18-year-olds Sex: 53.4 % girls	Average 15.6 days (range 10–22 days)	Watch ≤2 h. TV on average school day: Kappa 46.7 %	Good	−
Adolescent Sedentary Activity Questionnaire (ASAQ) [Brazilian version] [30]	*n* = 122 Age: 14.0 ± 1.4 years (12–17) Sex: 51 % girls	3 days	Total SB full week, 12- to 14-year-olds; ICC: boys 0.92 (95 % CI 0.74–0.92), girls 0.85 (95 % CI 0.64–0.93) Total SB full week, 15- to 17-year-olds; ICC: boys 0.89 (95 % CI 0.77–0.94), girls 0.93 (95 % CI 0.85–0.96) Total SB weekdays, 12- to 14-year-olds; ICC: boys 0.90 (95 % CI 0.80–0.95), girls 0.90 (95 % CI 0.80–0.95) Total SB weekdays, 15- to 17-year-olds; ICC: boys 0.73 (95 % CI 0.36–0.88), girls 0.89 (95 % CI 0.76–0.95) Total SB weekend, 12- to 14-year-olds; ICC: boys 0.84 (95 % CI 0.69–0.92), girls 0.73 (95 % CI 0.47–0.87) Total SB weekend, 15- to 17-year-olds; ICC: boys 0.84 (95 % CI 0.63–0.93), girls 0.58 (95 % CI 0.09–0.80)	Fair	+
Flemish Physical Activity Computer Questionnaire (FPACQ) [67]	*n* = 33 Age: 14.4 ± 1.4 years Sex: 70 % girls	9 days	Inactivity (TV and computer): ICC 0.83, kappa 0.61	Fair	+/−
Healthy Lifestyle Europe by Nutrition in Adolescence (HELENA) screen-time-based sedentary behavior questionnaire [68]	*n* = 183 Age: 12.5–17.5 years Sex: 57 % girls	1 week	TV viewing: Kappa: weekdays 0.71, weekend 0.68 Computer games: Kappa: weekdays 0.82, weekend 0.79 Console games: Kappa: weekdays 0.82, weekend 0.81; Internet non-study: Kappa: weekdays 0.86, weekend 0.71; Internet for study: Kappa: weekdays 0.46, weekend 0.33; Study: Kappa: weekdays 0.73, weekend 0.82	Fair	+/−
Measures of out-of-school sedentary and travel behaviors of the international Healthy Environments and active living in teenagers—Hong Kong [iHealt(H)] study [45]	*n* = 68 Age: 15.4 years Sex: 47 % girls	13 days (range 8–16 days)	Watching TV/DVD/video: ICC 0.62 Playing sedentary computer or video games: ICC 0.66 Using internet/emailing/other electronic media for leisure: ICC 0.58 Doing homework:^f^ ICC 0.78 Reading a book (not for school): ICC 0.61 Riding in a car, bus, etc.: ICC 0.51	Fair	−
The International Physical Activity Questionnaire (IPAQ) self-administered short version [31]	*n* = 71 Age: 14.9 years (13–18) Sex: 56.3 % girls	8–12 days	Sitting: ICC 0.27 (95 % CI −0.50 to 0.54)	Fair	−
Newly developed questionnaire on total sedentary time [69]	*n* = 20 Age: 15.4 ± 1.4 years Sex: 42 % girls	Mean: 16 ± 9 days	Total SB time: ICC: weekday 0.37 (95 % CI −0.09 to 0.70), weekend day 0.67 (95 % CI 0.32–0.86), average day 0.45 (95 % CI 0.01–0.74) Context-specific sedentary behaviors: ICC range (95 % CI −0.06 to 0.92), 8 % excellent, 13 % good, 42 % moderate, 38 % poor^g^	Poor	−

*ICC* intraclass correlation coefficient, *SCC* Spearman correlation coefficient, *PCC* Pearson correlation coefficient, *SB* sedentary behavior, *CI* confidence interval, *SD* standard deviation, *PC* personal computer, *COSMIN* COnsensus-based Standards for the selection of health Measurement INstruments, + indicates ≥80 % acceptable correlations, +/− indicates ≥50 % to <80 % acceptable correlations, − indicates <50 % acceptable correlations

^a^Age presented as mean age ± SD (range)

^b^Based on the COSMIN checklist

^c^Significant differences: watching TV on school days in girls and boys, and 11- and 15-year-olds: ICC 0.91, 0.51, 0.86, and 0.57, respectively; playing PC or console games at weekends in girls and boys: ICC 0.47 and 0.83, respectively; using the PC at weekends in 11- and 15-year-olds: ICC 0.83 and 0.16, respectively

^d^Two questionnaires combined

^e^Modified question of the YRBS

^f^Significant differences: doing homework, boys and girls: ICC 0.69 and 0.85, respectively

^g^Not based on our criteria

### Measurement Error

Table 3 shows an overview of the nine studies that assessed the measurement error of questionnaires. One of the included measurement error studies received a good methodological quality rating, while eight of the studies received a fair rating, predominantly due to the lack of describing how missing items were handled. The questionnaires showing the highest percentage of agreement between two measurements are the ‘Questionnaire for measuring length of sleep, television habits and computer habits’ [44], and the ‘Measures of out-of-school sedentary and travel behaviors of the iHealt(H) study’ [45], for children and adolescents, respectively.

**Table 3:** Measurement error of sedentary behavior questionnaires for youth, sorted by age category and methodological quality

Questionnaire	Study population^a^	Time interval	Results	Methodological quality^b^
Preschoolers (mean age <6 years)				
Preschool-aged Children’s Physical Activity Questionnaire (Pre-PAQ) [proxy] [48]	*n* = 103 Age: 3- to 5-year-olds Sex: 48 % girls	1–2 weeks	ME range from 5.5 min (time spent on the computer, weekend) to 13.8 min (time spent watching TV, week)	Good
Children (mean age ≥6 years and <12 years)				
Questionnaire for measuring length of sleep, TV habits and computer habits (proxy and non-proxy) [44]	*n* = 138 Age: 6-, 7-, 10-, 14- and 16-year olds Sex: 53 % girls	2 weeks	Time spent watching TV: PoA 75.5 % Time spent at a computer: PoA 84.6 %	Fair
UP4FUN child questionnaire [62]	*n* = 143 Age: 10- to 12-year-olds Sex: 57 % girls	1 week	TV/DVD watching: PoA: weekdays 53 %, weekend days 39 %, yesterday 40 % Computer/game console: PoA: weekdays 53 %, weekend days 39 %, yesterday 48 % Breaking up TV/DVD watching: PoA 47 % Breaking up computer/game console: PoA 48 % Breaking up school lesson: PoA 65 %	Fair
The Eating and Activity Questionnaire Trial (Project EAST) and a modified question of the Youth Risk Behavior Survey (YRBS)^c^ [51]	*n* = 245 Age: 11- to 15-year-olds Sex: 41 % girls	1 week	Weekday TV, school year:^d^ PoA: exact agreement 48.16; +1 category 86.94; percentage of children meeting recommendation of <2 h/day TV viewing 82.04 Weekend TV, school year: PoA: exact agreement 45.31; +1 category 81.22; percentage of children meeting recommendation of <2 h/day TV viewing 76.73 Weekday TV, summer: PoA: exact agreement 40.82; +1 category 74.69; percentage of children meeting recommendation of <2 h/day TV viewing 73.06 Weekend TV, summer: PoA: exact agreement 35.10; +1 category 74.69; percentage of children meeting recommendation of <2 h/day TV viewing 69.80 Computer use: PoA: exact agreement 50.20; +1 category 85.71; percentage of children meeting recommendation of <2 h/day TV viewing 82.45	Fair
The ENERGY-project Dietary and Physical Activity Habits of Children–child questionnaire [53]	*n* = 730; Age: (11.3 ± 0.5–12.5 ± 0.6 years) Sex: 47–58 % girls	1 week	TV watching: PoA: weekdays 42 %, weekend days 36 %, yesterday 36 % Computer use: PoA: weekdays 41 %, weekend days 38 %, yesterday 39 % Travel by car to school: PoA 84 % Transport today to school: PoA 83 % Travel by public transport to school: PoA 92 % Activity during breaks: PoA 86 %	Fair
Selected indicators from the Health Behavior in School-aged Children (HBSC) questionnaire [40]	*n* = 95 Age: 11.7 ± 0.4 years (*n* = 44)/15.8 ± 0.3 years (*n* = 51) Sex: 55 % girls (*n* = 44) 39 % girls (*n* = 51)	3 weeks	Playing PC or console games on school days: PoA 60 % Playing PC or console games at weekends: PoA 40 % Watching TV on school days: PoA 57 % Watching TV at weekends: PoA 38 % Doing homework on school days: PoA 56 % Doing homework at weekends: PoA 42 % Using PC on school days: PoA 41 % Using PC at weekends: PoA 32 %	Fair
Older children and adolescents (mean age ≥12 years)				
Measures of out-of-school sedentary and travel behaviors of the international Healthy Environments and active living in teenagers – Hong Kong [iHealt(H)] study [45]	*n* = 68 Age: 15.4 years Sex: 47 % girls	13 days (range 8–16 days)	Watching TV/DVD/video: PoA 74 % Playing sedentary computer or video games: PoA 72 % Using internet/emailing/other electronic media for leisure: PoA 65 % Doing homework:^e^ PoA 76 % Reading a book (not for school): PoA 62 % Riding in a car, bus, etc.: PoA 68 %	Fair
Adolescent Sedentary Activity Questionnaire (ASAQ) [Brazilian version] [30]	*n* = 122 Age: 14.0 ± 1.4 years (12–17) Sex: 51 % girls	3 days	Total sedentary time (min): MD: 116.6 (min); LoA [−1750 to 1980]	Fair
Flemish Physical Activity Computer Questionnaire (FPACQ) [67]	*n* = 33 Age: 14.4 ± 1.4 years Sex: 70 % girls	9 days	Inactivity (TV and computer): Proportion agreement 0.74	Fair

*MD* mean difference, *LoA* limits of agreement, *PoA* percentage of agreement, *ME* measurement error, *PC* personal computer, *COSMIN* COnsensus-based Standards for the selection of health Measurement INstruments

^a^Age presented as mean ± SD (range)

^b^Based on the COSMIN checklist

^c^Two questionnaires combined

^d^Modified question of the YRBS

^e^Significant differences: doing homework, boys and girls: PoA 67 and 88 %, respectively

### Internal Consistency

Internal consistency was analyzed in two of the included studies, demonstrating acceptable Cronbach’s alphas (i.e. 0.75 for the unidimensional sedentary lifestyle subscale [35], and 0.78 for the unidimensional sedentary behavior subscale [46]). The methodological quality was rated as good and excellent, respectively.

### Construct Validity

Of the included construct validity studies, 3 included preschoolers as a study population, 13 studies included children, and 6 studies included adolescents and older children. Table 4 summarizes the construct validity studies (*n* = 21) examining the relationship of the questionnaire with other measurement instruments. None of these studies received an excellent or good methodological quality rating, 5 received a fair rating, and 16 were rated as poor. Major reasons for the low methodological quality scores were both the lack of a priori formulated hypotheses and the use of comparison measures with unknown measurement properties. In preschoolers, the Direct Estimate [47] seemed the most valid, currently available, sedentary behavior questionnaire as it received a positive level 2 evidence rating and a fair methodological quality rating. In children, the Youth Activity Profile [52] seemed the most valid questionnaire as it received a positive level 2 evidence rating and a fair methodological quality. Studies in adolescents only received negative evidence ratings, thus no final conclusion regarding the most valid sedentary behavior questionnaires can be drawn. One of the construct validity studies was not included in Table 4 [46] as it examined construct validity by testing a hypothesis with regard to differences in scores between known groups. On the Energy Retention Behavior Scale, scores for known group validity demonstrated statistically significant higher scores for overweight or obese children than for underweight or normal-weight children, which was in line with the a priori hypothesis.

**Table 4:** Validity of sedentary behavior questionnaires for youth, sorted by age category, methodological quality, and level of evidence and evidence rating

Questionnaire	Study population^a^	Comparison measure		Results	Methodological quality^b^	Level of evidence and evidence rating^c^
Preschoolers (mean age <6 years)						
The Direct Estimate (proxy) [47]	*n* = 330	Daily activity chart; TV viewing diary (first correlated with TV observation (*n* = 105) *r* = 0.84–0.86 [including/excluding uncertainty])	TV viewing: Versus daily activity chart: *r* = 0.62 (direct estimate 22.0 h/wk vs. daily activity chart 17.7 h/wk) TV viewing: Versus diary: *r* = 0.60 (direct estimate 22.0 h/wk vs. diary 16.7*)		Fair	Level 3: − Level 2: +
Daily Activity Chart (proxy) [47]	*n* = 330	The Direct Estimate; TV viewing diary (first correlated with TV observation (*n* = 105) *r* = 0.84–0.86 [including/excluding uncertainty])	TV viewing: Versus direct estimate: *r* = 0.62 (direct estimate 22.0 h/wk vs. daily activity chart 17.7 h/wk) TV viewing: Versus diary: *r* = 0.48 (activity chart 17.7 h/wk vs. diary 16.7*)		Fair	Level 3: − Level 2: −
Physical activity and sedentary behavior questionnaire (based on the Canadian Health Measures Survey) [proxy] [28]	*n* = 87 Age: 4–70 months Sex: 54 % girls	Accelerometer (Actical)	Total SB: median difference 306 min/day*, LoA [125–460],^d^ SCC 0.10 (95 % CI −0.12 to −0.33) Screen time: SCC −0.05 (95 % CI −0.27 to 0.18) Stroller time: SCC 0.31 (95 % CI 0.09–0.50) Motor vehicle time: SCC −0.09 (95 % CI −0.30 to 0.13)		Poor	Level 2: −
Physical activity questionnaire for parents of preschoolers [49]	*n* = 35 Age: 4.4 ± 0.7 years (3–5) Sex: 51 % girls	Accelerometer (Actigraph GT1M)	Sirard sedentary cut point *r* = 0.35 Pate sedentary cut point *r* = 0.34		Poor	Level 2: −
Children (mean age ≥6 and <12 years)						
Youth Activity Profile (YAP) [52]	*n* = 161 Age: 9.7 ± 1.0 years, 11.7 ± 0.8 years, 15.7 ± 1.2 years (total sample) Sex: 56 % girls (total sample) *n* = 291 Age: 9.7 ± 1.0 years, 11.7 ± 0.8 years, 15.7 ± 1.2 years Sex: 56 % girls	Sense Wear Armband (SWA)	Sedentary time: PCC 0.75, MD −49.7 ± 23.1 min/wk, LoA (90%) [−88.0 to −11.4] YAP composite score for home sedentary sign. Correlated with SWA: *b* = 9.88 ± 2.40		Fair	Level 2: +
Children’s Leisure Activities Study Survey–Chinese version questionnaire (CLASS–C) [54]	*n* = 99 Age 9- to 12-year-olds Sex: 67 % girls	Accelerometer (Actigraph GT1M)	Sedentary time boys: SROC: weekdays 0.09, weekends −0.16, 1 week 0.06 Sedentary time girls: SROC: weekdays 0.19, weekends 0.18, 1 week 0.25		Fair	Level 2: −
Canadian Health Measures Survey (CHMS) [proxy] [27]	*n* = 878 Age: 8.7 years (6–11) Sex: 49 % girls	Accelerometer (Actical)	Sedentary/screen time: PCC 0.17		Poor	Level 2: −
TV viewing items of the Health Behavior in School-Aged Children survey (HBSC) [39]	*n* = 111 Age: 11.8 ± 0.6 years Sex: 48 % girls	TV viewing diary	Mean TV viewing: ICC: boys 0.36 (95 % CI 0.11–0.57), girls 0.54 (95 % CI 0.32–0.71) Significantly higher TV viewing for questionnaire vs. diary hours/day (SD): boys: 2.96 (1.84) for questionnaire, 1.91 (1.14) for diary; girls: 2.03 (1.25) for questionnaire, 1.43 (0.89) for diary		Poor	Level 2: −
Parent proxy-report of physical activity and sedentary activities (proxy) [63]	*n* = 167 (validity vs. accelerometer); 125 (validity vs. diary) Age: 6- to 10-year-olds; 13- to 14-year-olds Sex: 51 % girls (in total sample *n* = 189)	Accelerometer (Actigraph model AM7164) and time–activity diary (physical activity record)	Versus accelerometer: SCC (adjusted for school, sex, grade, maternal education): overall sedentary activities 0.55 (0.01); TV/DVD/video watching 0.32 (0.00); sitting at a computer/playing Nintendo/electronic games 0.32 (−0.03); doing homework 0.53 (0.03); reading 0.32 (−0.06); playing a musical instrument 0.12 (−0.01); playing quiet/other activities −0.10 (0.01); traveling by car/public transport 0.05 (−0.03) Versus. diary: an increase in mean questionnaire-reported sedentary time paralleled an increase in mean diary-reported SB		Poor	Level 2: −
The Eating and Activity Questionnaire Trial (Project EAST) and a modified question of the Youth Risk Behavior Survey (YRBS)^e^ [51]	*n* = 245 Age: 11- to 15-year-olds Sex: 41 % girls	TV and computer logs	Weekend TV: MD (SD) −0.21 (2.54), SCC 0.366 Weekly average TV: MD (SD) −0.09 (1.75), SCC 0.466 Computer only: MD (SD) 0.68 (1.26), SCC 0.394 Weekday TV:^f^ MD (SD) −0.04 (1.70), SCC 0.457		Poor	Level 2: −
Self-Administered Physical Activity Checklist (SAPAC) [38]	*n* = 125 Age: 10.9 ± 0.5 years Sex: 56 % girls	Physical activity checklist interview (PACI)	Time in sedentary activities: ICC 0.75; MD (SE): 15 (7)		Poor	Level 3: +
The ENERGY-project Dietary and Physical Activity Habits of Children–child questionnaire [53]	*n* = 96; Age (11.4 ± 0.6–12.0 ± 0.6 years) Sex: 31–67 % girls	Cognitive interview	TV watching: ICC: weekdays 0.63, weekend days 0.56, yesterday 0.70 Computer use: ICC: weekdays 0.35, weekend days 0.65, yesterday 0.28 Travel by car to school: ICC 0.84 Transport today to school: ICC 0.67 Travel by public transport to school: ICC 0.81 Activity during breaks: ICC 0.65		Poor	Level 3: −
Sedentary behavior items from a new questionnaire to identify usual patterns of physical activity [33]	*n* = 69 Age: 9.9 years (8.5–12.7) Sex: 100 % girls	1-week activity diaries	Sitting, school day: ICC: girls 0.40, mothers 0.03, fathers 0.04 Sitting, weekend day: ICC: girls 0.32, mothers 0.15, fathers 0.10 TV, school day: ICC: girls 0.38, mothers 0.54, fathers 0.52 TV, weekend day: ICC: girls 0.31, mothers 0.31, fathers 0.40 Sitting, school day hours/day (SD): diary 7.6 (2.0), daughters 7.3 (2.1), mothers 6.2 (2.1)*, fathers 6.0 (2.7)* Sitting, weekend day hours/day (SD): diary 6.7 (2.3), daughters 6.1 (1.7)*, mothers 4.9 (1.8)*, fathers 4.7 (2.3)* TV, school day hours/day (SD): diary 1.2 (1.4), daughters 2.6 (2.1)*, mothers 1.4 (1.2), fathers 1.5 (1.2)* TV, weekend day hours/day (SD): diary 2.0 (2.2), daughters 4.1 (3.4)*, mothers 2.6 (1.6)*, fathers 2.5 (1.6)*		Poor	Level 3: −
HABITS questionnaire [70]	*n* = 35 Age: 11.8 ± 2.3 years Sex: 37 % girls	Modifiable Activity Questionnaire	Watching TV, weekday: SROC 0.56 Watching TV, weekend day: SROC 0.59		Poor	Level 3: −
Questions from the National Longitudinal Survey of Children and Youth (proxy) [71]	*n* = 3925 parents and children (grade 5) [TV viewing question] *n* = 3955 parents and children (grade 5) [computer use and video games question]	Parent-reported questions from the National Longitudinal Survey of Children and Youth	TV viewing: Kappa 0.19 (95 % CI 0.16–0.21) Computer use and video games: Kappa 0.23 (95 % CI 0.20–0.25)		Poor	Level 3: −
Sedentary Behavior and Sleep Scale (SBSS) [43]	*n* = 45 (weekend), 54 (weekday) Age: 6.7 ± 0.5 years (total sample) Sex: 59 % girls (total sample)	Accelerometer (RT3, Stayhealthy)	Sedentary time: weekday MD 79 ± 113 min/day, LoA [−143.6 to 303.3], weekend MD 400 min/day, LoA [−120.2 to 920.8] Bland–Altman plot, weekend, depicts a positive magnitude bias^g^		Poor	Level 3: ?
Older children and adolescents (mean age ≥12 years)						
Modified 3-day Self-Administered Physical Activity Checklist (SAPAC) [36]	*n* = 190 Age: 11- to 15-year-olds Sex: 64 % girls	Accelerometer (Actigraph, formerly CSA accelerometer model 7164)	Overall SB [adjusted for total minutes of activity]: PCC 0.18 (95 % CI 0.07–0.28) [0.23 (95 % CI 0.12–0.33)] SCC 0.14 (95 % CI 0.05–0.23) [0.21 (95 % CI 0.12–0.30)]		Fair	Level 2: −
Activity Questionnaire for Adults and Adolescents (AQuAA) [65]	*n* = 42 Age: 13.4 ± 1.0 years Sex: 50 % girls	Accelerometer (Actigraph model 7164)	Sedentary activities: SCC 0.23; two hypotheses/one hypotheses confirmed		Fair	Level 3: −
Newly developed questionnaire on total sedentary time [69]	*n* = 62 Age: 16.1 ± 1.1 years Sex: 58 % girls	Movement monitor (activPAL)	SB time: SROC: weekday 0.42 (95 % CI 0.19–0.61); weekend day 0.02 (95 % CI −0.23 to 0.27); average day 0.29 (95 % CI 0.04–0.50) SB time: MD: weekday 57.05 %, weekend day 46.29 %, average day 53.34 % Bland–Altman plot, weekend, depicts a small negative magnitude bias^h^		Poor	Level 1: −
International Physical Activity Questionnaire–Short Form (IPAQ-SF) [Chinese version] [32]	*n* = 1021 Age: 14.3 ± 1.6 years Sex: 47 % girls	Accelerometer (ActiGraph GT3X+ or GT3X)	Sitting: SCC: all 0.18, boys 0.24, girls 0.10		Poor	Level 2: −
Healthy Lifestyle Europe by Nutrition in Adolescence (HELENA) screen-time-based sedentary behavior questionnaire [68]	*n* = 2048 adolescents Age: 12.5–17.5 years Sex: percentage girls unknown	Accelerometer (Uni-axial, Actigraph MTI, model GT1M)	Median percentage (25th–75th percentile) of objectively measured SB time across tertiles of self-reported SB:^i^ Boys: computer games: tertile 1, 79.7 (76.2–83.6); tertile 2, 79.8 (77.0–83.6); tertile 3, 81.1 (77.6–85.2) [1–3; 2–3*] Internet non-study: tertile 1, 79.6 (76.0–83.6); tertile 3, 81.0 (77.9–84.6) [1–3*] Internet for study: tertile 1, 79.3 (75.7–83.3); tertile 2, 81.1 (78.2–84.8); tertile 3, 80.6 (77.3–84.5) [1–2; 1–3*] Study: tertile 1, 79.6 (76.2–83.8); tertile 2, 80.3 (77.0–84.0); tertile 3, 81.2 (78.2–84.7) [1–3; 2–3*] Total SB weekdays: tertile 1, 79.9 (76.8–83.4); tertile 2, 80.8 (77.3–84.9); tertile 3, 80.7 (77.4–84.6) [1–2; 1–3*] Total SB weekend: tertile 1, 79.5 (76.9–83.1); tertile 2, 81.1 (77.9–85.0); tertile 3, 81.0 (78.1–84.6) [1–2; 1-3*] Girls: Study: tertile 1, 82.4 (80.0–85.1); tertile 3, 83.1 (80.8–86.1) [1–3*]		Poor	Level 2: ?
A questionnaire to measure a broad range of sedentary activities [72]	*n* = 172 Age: 12.8 years (12–15) Sex: 100 % girls	Accelerometer (MTI)	SB: MD weekly (SD): −3.2 h/wk (11.9), LoA [−26.5 to 20.1] Bland–Altman plot depicts a small positive magnitude bias^j^		Poor	Level 2: ?

*SB* sedentary behavior, *MD* mean difference, *LoA* limits of agreement, *PCC* Pearson correlation coefficient, *SROC* Spearman rank order correlation, *SCC* Spearman correlation coefficient, *r* correlation coefficient, *CI* confidence interval, *COSMIN* COnsensus-based Standards for the selection of health Measurement INstruments, *ICC* intraclass correlation coefficient, *SD* standard deviation, * indicates significant, ? indicates evidence rating unclear due to a lack of hypotheses

^a^Age presented as mean age ± SD (range)

^b^Based on the COSMIN checklist

^c^Based on Table 1: + indicates ≥80 % acceptable correlations; +/− indicates ≥50 to <80 % acceptable correlations; − indicates <50 % acceptable correlations

^d^Estimation, derived from the Bland–Altman plot

^e^Two questionnaires combined

^f^Modified question of the YRBS

^g^Bland–Altman plot indicates larger differences between self-report and objective measures as the mean sedentary time increases (no statistical analysis used)

^h^Bland–Altman plot indicates smaller differences between self-report and objective measures as the objectively measured sedentary time increases (no statistical analysis used)

^i^Significant differences between tertiles, indicating appropriate ranking of self-reported sedentary behavior

^j^Bland–Altman plot indicates larger differences between self-report and objective measures as the mean sedentary time decreases and increases (no statistical analysis used)

### Structural Validity

Two of the included studies analyzed the structural validity of the questionnaire, i.e. the Korean Youth Risk Behavior Survey (KYRBS) [35] and the Energy Retention Behavior Scale for Children (ERB–C scale) [46]. Structural validity was assessed by performing confirmatory factor analysis. The KYRBS includes five subscales, including one sedentary lifestyle subscale, while the ERB–C scale includes two subscales, one of which is sedentary behavior. Both studies showed acceptable fit of the expected factor structures, i.e. Normed Fit Index (NFI) 0.960, Turker–Lewis Index (TLI) 0.956, CFI 0.969 and root mean squared error of approximation (RMSEA) 0.034 for the KYRBS [35], and NFI 0.91, non-NFI (NNFI) 0.92, CFI 0.95, and RMSEA 0.08 for the ERB–C scale [46]. The methodological quality was rated as good and excellent, respectively.

### Content Validity

Eight studies evaluated the content validity of the questionnaire, of which four predominantly focused on the comprehensibility of the questionnaire by asking children or parents about, for example, terminology, appropriateness of reading level, ambiguity, and other difficulties [29, 44, 46, 48]. The other four studies focused on the content of the questionnaire by consulting experts, e.g. researchers active in the field of physical activity, about, for example, relevance of items [30, 44, 46, 48]. Due to the minimal information about the procedures available in the greater part of the included studies, it was impossible to assess the quality of the content validity studies and to thus interpret the results. In addition, in seven of the included studies, pilot testing of the questionnaire for comprehensibility was incorporated. Unfortunately, too little information was provided to assess the methodology of the content validity examination [33, 38, 45, 49–52]. Additionally, translation processes were mentioned in six [24, 30, 42, 45, 53, 54] of the included studies. Due to minimal information about the methods used, the quality of the greater part of these studies was unclear.

## Discussion

The aim of this review was to summarize existing evidence on the measurement properties of self-report or proxy-report questionnaires assessing sedentary behavior in children and adolescents under the age of 18 years. Additionally, we summarized the characteristics of the included self-report and proxy-report questionnaires. Our summary yielded a wide variety of questionnaires, designed for different target populations and assessing different constructs and dimensions of sedentary behavior. Test–retest reliability correlations of the included questionnaires ranged from 0.06 to 0.97. In addition, correlations found for construct validity ranged from −0.16 to 0.84. Although a number of studies received a positive evidence rating for test–retest reliability or construct validity, the methodological quality of the studies was mostly rated as fair or poor. Unfortunately, no questionnaires assessing total sedentary behavior or other constructs of sedentary behavior with both a positive evidence rating for reliability and validity were available. Hence, we have no conclusive recommendation about the best available sedentary behavior self-report or proxy-report questionnaire in children and adolescents.

### Reliability and Measurement Error

As the methodological quality of the included studies assessing test–retest reliability and/or measurement error was mainly rated as fair or poor, no definite conclusion can be drawn about the reliability of the majority of the examined sedentary behavior questionnaires. Moreover, the lack of multiple studies assessing the same questionnaire in the same target population further limited the ability to draw final conclusions. To achieve higher methodological quality for both reliability and measurement error, we recommend that future studies include detailed descriptions of the methods used, e.g. how missing items were handled, and to include an appropriate sample size [15, 17]. Additionally, as correlations varied across different recall periods (e.g. usually, or yesterday), and different time frames and constructs of sedentary behavior (e.g. weekdays and weekend days, overall sedentary behavior, and watching television), no conclusion can be drawn about specific time frames or constructs of sedentary behavior being more reliable than others. Additionally, when measurement errors occur, information on the MIC should be available to allow interpretation of the results [21]. To the best of our knowledge, no information on the MIC is available as yet.

### Construct Validity

Due to the low methodological quality of the included studies examining validity, and the lack of multiple studies assessing the same questionnaire, no conclusive conclusion can be drawn about the validity of the examined questionnaires. We specifically recommend future validity studies to describe a priori hypotheses, and choose comparison measures with known and acceptable measurement properties. The low methodological quality of all included validity studies might partly explain the high prevalence of negative evidence ratings, i.e. <50 % acceptable correlations.

Studies demonstrating acceptable correlations often used comparison measures providing weaker levels of evidence, i.e. other questionnaires or cognitive interviews (level 3 evidence). In general, higher correlations were found when lower levels of evidence comparison measures were used. A possible explanation might be the equivalence of dependence on recall in both the questionnaire under study and the comparison measure, i.e. other questionnaires or cognitive interviews, compared with objective, higher levels of evidence comparison measures, e.g. inclinometers and accelerometers. Other potential factors that may explain the low correlations may be inadequate content validity, the lack of a gold standard, and a mismatch in time frames between questionnaire and comparison measures. As the studies lack information about the development of the questionnaires (e.g. a justification of the constructs included, and the dimensions measured), and lack appropriate testing of the relevance, comprehensiveness, and comprehensibility of the content of the questionnaires, it remains unclear whether the content validity of the included questionnaires is acceptable. Evaluating the content validity of questionnaires is essential to obtaining insight into the comprehensibility of the questionnaire for the target population, and to ensure all relevant aspects of the construct are measured and that no irrelevant aspects are included [20]. Without evaluating these aspects of validity, there is no certainty the questionnaire measures what it is supposed to measure. The limited attention to content validity is also shown by the wide variety of constructs (e.g. watching television, quiet play, studying), and dimensions (e.g. duration and frequency) being measured by the included questionnaires. A justification of these choices is lacking. Only two studies, by Tucker et al. [23, 24], provided sufficient description and support for the development of their questionnaire, e.g. experts of the field and the target population were consulted and contributed to the content of the questionnaire.

Furthermore, studies using a translated version of an existing questionnaire often did not report sufficient information about the translation processes. Only the studies by de Fátima Guimarães et al. [30] and Tucker et al. [24] included adequate descriptions of the translation process, e.g. translations by language experts, and review by experts in the field. Moreover, cross-cultural validation of the translated questionnaires was often not conducted, making it impossible to examine whether the questionnaire truly measured the same constructs as the original questionnaire [22].

Additionally, the available objective measures of sedentary behavior, e.g. inclinometers or accelerometers, are still subject to subjectivity, e.g. the definition of non-wear time, the minimum number of valid hours per day and number of valid days, and the selection of a cut point for sedentary behavior remain subjective decisions. The accelerometer cut points for sedentary behavior in the included studies varied from <100 to <699 cpm, leading to different estimates of sedentary time. Importantly, constructs measured by questionnaire and accelerometer may not correspond when cut points deviating from <100 cpm are applied [55] as measured constructs may not match, i.e. they may exclude parts of sedentary time or include light physical activity, respectively. The problem of mismatched constructs also occurs in some cases due to non-corresponding time frames addressed by the measurement instrument and the comparison measures, e.g. leisure time versus all day.

### Strengths and Limitations

A major strength of our review is that the methodological quality rating was performed separately from the interpretation of the findings. This makes the final evidence rating more transparent, e.g. whether negative evidence ratings are due to low-quality questionnaires in case of good or excellent methodological quality studies, or may be biased, in case of poor methodological quality. Additionally, through structured cross-reference searches, we also included studies that were not primarily aimed at examining measurement properties. Another strength is that at least two independent authors conducted the literature search and data extraction, as well as the quality rating. However, our review also has limitations. As most included studies did not report all details needed for an adequate quality rating, the quality ratings of the studies may have been underestimated. We did not contact authors for additional information as this would favor recent studies over older studies, thereby optimizing quality ratings of recent papers. Furthermore, only English-language papers were included, and as a result we might have missed relevant studies. Moreover, in some studies that were found through cross-reference searches, examining the measurement properties was not the primary aim. There is a possibility that not all such studies were found through cross-reference searches, yet finding these studies through systematic literature searches seems impossible as information on the assessment of measurement properties or sedentary behavior assessment by the questionnaires is lacking in the titles and abstracts.

### Recommendations for Future Studies

Studies focusing on the development of questionnaires need to pay more attention to content validity. Moreover, the content validity of currently available questionnaires needs to be examined by testing the relevance, comprehensiveness, and comprehensibility of the content of the questionnaires, using appropriate qualitative methods [22]. The COSMIN group is currently developing detailed standards for assessing content validity of health status questionnaires, which may also be useful for assessing content validity of sedentary behavior questionnaire (see http://www.cosmin.nl for more information). Criteria that, in our opinion, need to be considered are (i) a clear description and adequate reflection of the construct to be measured; (ii) comprehensibility of questions; (iii) appropriate response options; (iv) appropriate recall period; (v) appropriate mode of administration; and (vi) an appropriate scoring algorithm. A justification of choices needs to be provided, for example based on input from experts in the field and the target population.

More high-quality research on construct validity, reliability, measurement error, and responsiveness of the questionnaire is also needed, as well as studies on internal consistency and structural validity for questionnaires where this is applicable. To acquire high methodological quality studies, we recommend using a standardized tool, e.g. the COSMIN checklist [16, 56]. This tool can be used for the design of the study and provides an overview of what should be reported. Additionally, we recommend that when reviewers and journal editors evaluate studies, they take into consideration whether the investigators used such a standardized tool in order to prevent publishing of studies with inadequate information and low methodological quality. This need for a standardized tool for the assessment of measurement properties is consistent with recommendations by Kelly et al. [57].

In addition, for the construct validity of questionnaires assessing total sedentary time, we recommend using more objective, high-level evidence, comparison measures with available and acceptable measurement properties, e.g. inclinometers or accelerometers, instead of using measurement instruments with unknown or unacceptable measurement properties. Furthermore, appropriate accelerometer cut points for sedentary behavior need to be applied, e.g. <100 cpm [55, 58]. However, as the accuracy of accelerometers for measuring sedentary behavior remains questionable, and distinguishing sitting from standing quietly remains problematic [11], we recommend using the activPAL as an objective comparison measure for total sedentary time [9]. Importantly, the questionnaire in use and the comparison measure need to measure corresponding constructs and/or time frames. Additionally, stating a priori hypotheses should be carried out at all times to ensure unbiased interpretation of the results.

Finally, as a wide variety of questionnaires are available, we recommend researchers to critically review whether existing or slightly modified questionnaires are adequate for use in new studies, instead of developing new questionnaires. Moreover, we recommend authors of papers on measurement properties include the questionnaire under study and provide more details about its characteristics, e.g. questions and response options, so that researchers can assess whether existing questionnaires are adequate for their research.

## Conclusions

None of the self- or proxy-report sedentary behavior questionnaires for children and adolescents included in this review were considered both valid and reliable. Whether this is due to the low methodological quality of the included studies or to poorly developed questionnaires is unclear. In addition, the lack of multiple studies assessing both the validity and reliability of a questionnaire in the same study population also hampered our ability to draw a definite conclusion on the best available instruments. Therefore, we recommend more high-quality studies examining the measurement properties of the most promising sedentary behavior questionnaires. Acquiring high methodological quality can be obtained by using standardized tools such as the COSMIN checklist [16].

## Electronic supplementary material

Below is the link to the electronic supplementary material.

Supplementary material 1 (DOCX 139 kb)

Supplementary material 2 (DOCX 156 kb)
