# Supplementary material for: Systematic Review of Childhood Sedentary Behavior Questionnaires: What do We Know and What is Next?
Source: Sports Med. 2016 Aug 31;47(4):677–99. doi: 10.1007/s40279-016-0610-1 (PMC5357243; doi:10.1007/s40279-016-0610-1)
Supplement: Supplementary file 1 — Supplementary material 1 (DOCX 139 kb) [file 40279_2016_610_MOESM1_ESM.docx]

Electronic Supplementary Material Appendix S1. Search strategy

A systematic review of childhood sedentary behavior questionnaires: what do we know and what’s next?

Journal: Sports Medicine

Lisan M. Hidding,^1^ Teatske M. Altenburg,^1^ Lidwine B. Mokkink,^2^ Caroline B. Terwee,^2^ Mai J. M. Chin A Paw^1^

^1^Department of Public and Occupational Health, EMGO Institute for Health and Care Research, VU University Medical Center, Amsterdam, the Netherlands

^2^Department of Epidemiology and Biostatistics, EMGO Institute for Health and Care Research, VU University Medical Center, Amsterdam, the Netherlands

E-mail corresponding author: [l.hidding@vumc.nl](mailto:l.hidding@vumc.nl)

PubMed search strategy:

“sedentary behaviour”[tiab] OR “sedentary behaviours”[tiab] OR “sedentary behavior”[tiab] OR “sedentary behaviors”[tiab] OR “sedentary time”[tiab] OR “sedentary lifestyle”[tiab] OR “sitting time”[tiab] OR “prolonged sitting”[tiab] OR “TV time”[tiab] OR “TV viewing”[tiab] OR “watching TV” OR “computer time”[tiab] OR “computer use”[tiab] OR “screen time”[tiab] OR “screen-time”[tiab] OR “sedentary activity”[tiab] OR “sedentary activities”[tiab] OR “television”[tiab] OR “gaming” [tiab]

AND

instrumentation[sh] OR methods[sh] OR Validation Studies[pt] OR Comparative Study[pt] OR "psychometrics"[MeSH] OR psychometr*[tiab] OR clinimetr*[tw] OR clinometr*[tw] OR "outcome assessment (health care)"[MeSH] OR outcome assessment[tiab] OR outcome measure*[tw] OR "observer variation"[MeSH] OR observer variation[tiab] OR "Health Status Indicators"[Mesh] OR "reproducibility of results"[MeSH] OR reproducib*[tiab] OR "discriminant analysis"[MeSH] OR reliab*[tiab] OR unreliab*[tiab] OR valid*[tiab] OR coefficient[tiab] OR homogeneity[tiab] OR homogeneous[tiab] OR "internal consistency"[tiab] OR (cronbach*[tiab] AND (alpha[tiab] OR alphas[tiab])) OR (item[tiab] AND (correlation*[tiab] OR selection*[tiab] OR reduction*[tiab])) OR agreement[tiab] OR precision[tiab] OR imprecision[tiab] OR "precise values"[tiab] OR test-retest[tiab] OR (test[tiab] AND retest[tiab]) OR (reliab*[tiab] AND (test[tiab] OR retest[tiab])) OR stability[tiab] OR interrater[tiab] OR inter-rater[tiab] OR intrarater[tiab] OR intra-rater[tiab] OR intertester[tiab] OR inter-tester[tiab] OR intratester[tiab] OR intra-tester[tiab] OR interobserver[tiab] OR inter-observer[tiab] OR intraobserver[tiab] OR intra-observer[tiab] OR intertechnician[tiab] OR inter-technician[tiab] OR intratechnician[tiab] OR intra-technician[tiab] OR interexaminer[tiab] OR inter-examiner[tiab] OR intraexaminer[tiab] OR intra-examiner[tiab] OR interassay[tiab] OR inter-assay[tiab] OR intraassay[tiab] OR intra-assay[tiab] OR interindividual[tiab] OR inter-individual[tiab] OR intraindividual[tiab] OR intra-individual[tiab] OR interparticipant[tiab] OR inter-participant[tiab] OR intraparticipant[tiab] OR intra-participant[tiab] OR kappa[tiab] OR kappa's[tiab] OR kappas[tiab] OR repeatab*[tiab] OR ((replicab*[tiab] OR repeated[tiab]) AND (measure[tiab] OR measures[tiab] OR findings[tiab] OR result[tiab] OR results[tiab] OR test[tiab] OR tests[tiab])) OR generaliza*[tiab] OR generalisa*[tiab] OR concordance[tiab] OR (intraclass[tiab] AND correlation*[tiab]) OR discriminative[tiab] OR "known group"[tiab] OR factor analysis[tiab] OR factor analyses[tiab] OR dimension*[tiab] OR subscale*[tiab] OR (multitrait[tiab] AND scaling[tiab] AND (analysis[tiab] OR analyses[tiab])) OR item discriminant[tiab] OR interscale correlation*[tiab] OR error[tiab] OR errors[tiab] OR "individual variability"[tiab] OR (variability[tiab] AND (analysis[tiab] OR values[tiab])) OR (uncertainty[tiab] AND (measurement[tiab] OR measuring[tiab])) OR "standard error of measurement"[tiab] OR sensitiv*[tiab] OR responsive*[tiab] OR ((minimal[tiab] OR minimally[tiab] OR clinical[tiab] OR clinically[tiab]) AND (important[tiab] OR significant[tiab] OR detectable[tiab]) AND (change[tiab] OR difference[tiab])) OR (small*[tiab] AND (real[tiab] OR detectable[tiab]) AND (change[tiab] OR difference[tiab])) OR meaningful change[tiab] OR "ceiling effect"[tiab] OR "floor effect"[tiab] OR "Item response model"[tiab] OR IRT[tiab] OR Rasch[tiab] OR "Differential item functioning"[tiab] OR DIF[tiab] OR "computer adaptive testing"[tiab] OR "item bank"[tiab] OR "cross-cultural equivalence"[tiab]

AND

child*[tw] OR schoolchild*[tw] OR infan*[tw] OR adolescen*[tw] OR pediatri*[tw] OR paediatr*[tw] OR neonat*[tw] OR boy[tw] OR boys[tw] OR boyhood[tw] OR girl[tw] OR girls[tw] OR girlhood[tw] OR youth[tw] OR youths[tw] OR baby[tw] OR babies[tw] OR toddler*[tw] OR "Mental Disorders Diagnosed in Childhood"[MeSH] OR teen[tw] OR teens[tw] OR teenager*[tw] OR newborn*[tw] OR postneonat*[tw] OR postnat*[tw] OR puberty[tw] OR preschool*[tw] OR suckling*[tw] OR picu[tw] OR nicu[tw] OR "Arthritis, Juvenile Rheumatoid"[Mesh] OR "Myoclonic Epilepsy, Juvenile"[Mesh] OR "Leukemia, Myelomonocytic, Juvenile"[Mesh] OR "Xanthogranuloma, Juvenile"[Mesh] OR "Juvenile Delinquency"[Mesh] OR "Corneal Dystrophy, Juvenile Epithelial of Meesmann"[Mesh]

NOT

(addresses[PT] OR biography[PT] OR case reports[PT] OR comment[PT] OR directory[PT] OR editorial[PT] OR festschrift[PT] OR interview[PT] OR lectures[PT] OR legal cases[PT] OR legislation[PT] OR letter[PT] OR news[PT] OR newspaper article[PT] OR patient education handout[PT] OR popular works[PT] OR congresses[PT] OR consensus development conference[PT] OR consensus development conference, nih[PT] OR practice guideline[PT]) OR (animals[MeSH Terms] NOT humans[MeSH Terms])

EMBASE search strategy:

'sedentary lifestyle'/exp OR ‘sedentary lifestyle’:ab,ti OR ‘sitting’/exp OR ‘sitting time’:ab,ti OR ‘prolonged sitting’:ab,ti OR ‘sedentary behaviour’:ab,ti OR ‘sedentary behaviours’:ab,ti OR ‘sedentary behavior’:ab,ti OR ‘sedentary behaviors’:ab,ti OR ‘sedentary time’:ab,ti OR ‘television’:ab,ti OR ‘TV time’:ab,ti OR ‘TV viewing’:ab,ti OR ‘watching TV’:ab,ti OR ‘computer time’:ab,ti OR ‘computer use’:ab,ti OR ‘screen time’:ab,ti OR ‘screen-time’:ab,ti OR ‘sedentary activity’:ab,ti OR ‘sedentary activities’:ab,ti OR ‘gaming’:ab,ti

AND

'intermethod comparison'/exp OR 'data collection method'/exp OR 'validation study'/exp OR 'feasibility study'/exp OR 'pilot study'/exp OR 'psychometry'/exp OR 'reproducibility'/exp OR reproducib*:ab,ti OR 'audit':ab,ti OR psychometr*:ab,ti OR clinimetr*:ab,ti OR clinometr*:ab,ti OR 'observer variation'/exp OR 'observer variation':ab,ti OR 'discriminant analysis'/exp OR 'validity'/exp OR reliab*:ab,ti OR valid*:ab,ti OR 'coefficient':ab,ti OR 'internal consistency':ab,ti OR (cronbach*:ab,ti AND ('alpha':ab,ti OR 'alphas':ab,ti)) OR 'item correlation':ab,ti OR 'item correlations':ab,ti OR 'item selection':ab,ti OR 'item selections':ab,ti OR 'item reduction':ab,ti OR 'item reductions':ab,ti OR 'agreement':ab,ti OR 'precision':ab,ti OR 'imprecision':ab,ti OR 'precise values':ab,ti OR 'test-retest':ab,ti OR ('test':ab,ti AND 'retest':ab,ti) OR (reliab*:ab,ti AND ('test':ab,ti OR 'retest':ab,ti)) OR 'stability':ab,ti OR 'interrater':ab,ti OR 'inter-rater':ab,ti OR 'intrarater':ab,ti OR 'intra-rater':ab,ti OR 'intertester':ab,ti OR 'inter-tester':ab,ti OR 'intratester':ab,ti OR 'intratester':ab,ti OR 'interobeserver':ab,ti OR 'inter-observer':ab,ti OR 'intraobserver':ab,ti OR 'intraobserver':ab,ti OR 'intertechnician':ab,ti OR 'inter-technician':ab,ti OR 'intratechnician':ab,ti OR 'intratechnician':ab,ti OR 'interexaminer':ab,ti OR 'inter-examiner':ab,ti OR 'intraexaminer':ab,ti OR 'intraexaminer':ab,ti OR 'interassay':ab,ti OR 'inter-assay':ab,ti OR 'intraassay':ab,ti OR 'intra-assay':ab,ti OR 'interindividual':ab,ti OR 'inter-individual':ab,ti OR 'intraindividual':ab,ti OR 'intra-individual':ab,ti OR 'interparticipant':ab,ti OR 'inter-participant':ab,ti OR 'intraparticipant':ab,ti OR 'intraparticipant':ab,ti OR 'kappa':ab,ti OR 'kappas':ab,ti OR 'coefficient of variation':ab,ti OR repeatab*:ab,ti OR (replicab*:ab,ti OR 'repeated':ab,ti AND ('measure':ab,ti OR 'measures':ab,ti OR 'findings':ab,ti OR 'result':ab,ti OR 'results':ab,ti OR 'test':ab,ti OR 'tests':ab,ti)) OR generaliza*:ab,ti OR generalisa*:ab,ti OR 'concordance':ab,ti OR ('intraclass':ab,ti AND correlation*:ab,ti) OR 'discriminative':ab,ti OR 'known group':ab,ti OR 'factor analysis':ab,ti OR 'factor analyses':ab,ti OR 'factor structure':ab,ti OR 'factor structures':ab,ti OR 'dimensionality':ab,ti OR subscale*:ab,ti OR 'multitrait scaling analysis':ab,ti OR 'multitrait scaling analyses':ab,ti OR 'item discriminant':ab,ti OR 'interscale correlation':ab,ti OR 'interscale correlations':ab,ti OR ('error':ab,ti OR 'errors':ab,ti AND (measure*:ab,ti OR correlat*:ab,ti OR evaluat*:ab,ti OR 'accuracy':ab,ti OR 'accurate':ab,ti OR 'precision':ab,ti OR 'mean':ab,ti)) OR 'individual variability':ab,ti OR 'interval variability':ab,ti OR 'rate variability':ab,ti OR 'variability analysis':ab,ti OR ('uncertainty':ab,ti AND ('measurement':ab,ti OR 'measuring':ab,ti)) OR 'standard error of measurement':ab,ti OR sensitiv*:ab,ti OR responsive*:ab,ti OR ('limit':ab,ti AND 'detection':ab,ti) OR 'minimal detectable concentration':ab,ti OR interpretab*:ab,ti OR (small*:ab,ti AND ('real':ab,ti OR 'detectable':ab,ti) AND ('change':ab,ti OR 'difference':ab,ti)) OR 'meaningful change':ab,ti OR 'minimal important change':ab,ti OR 'minimal important difference':ab,ti OR 'minimally important change':ab,ti OR 'minimally important difference':ab,ti OR 'minimal detectable change':ab,ti OR 'minimal detectable difference':ab,ti OR 'minimally detectable change':ab,ti OR 'minimally detectable difference':ab,ti OR 'minimal real change':ab,ti OR 'minimal real difference':ab,ti OR 'minimally real change':ab,ti OR 'minimally real difference':ab,ti OR 'ceiling effect':ab,ti OR 'floor effect':ab,ti OR 'item response model':ab,ti OR 'irt':ab,ti OR 'rasch':ab,ti OR 'differential item functioning':ab,ti OR 'dif':ab,ti OR 'computer adaptive testing':ab,ti OR 'item bank':ab,ti OR 'cross-cultural equivalence':ab,ti

AND

AND ([newborn]/lim OR [infant]/lim OR [child]/lim OR [preschool]/lim OR [school]/lim OR [adolescent]/lim OR [young adult]/lim)

SPORTDiscus search strategy

TI sedentary lifestyle OR TI sedentary behaviour OR TI sedentary behavior OR AB sedentary lifestyle OR AB sedentary behaviour OR AB sedentary behavior OR AB prolonged sitting OR AB sitting time OR AB tv time OR AB tv viewing OR AB watching television OR AB viewing habits OR AB viewing time OR AB computer use OR AB computer time OR AB screen time OR AB television OR AB gaming OR TI prolonged sitting OR TI sitting time OR TI tv time OR TI tv viewing OR TI watching television OR TI viewing habits OR TI viewing time OR TI computer use OR TI computer time OR TI screen time OR TI television OR TI gaming

AND

TI schoolchild OR TI schoolchildren OR TI children OR TI infant OR TI ( infants or toddlers or young children ) OR TI neonatal OR TI ( adolescents or teenagers or young adults ) OR TI neonate OR TI ( paediatrics or children ) OR TI ( paediatrics or children or child or young person ) OR TI ( boys or girls ) OR TI boyhood OR AB schoolchild OR AB schoolchildren OR AB children OR AB infant OR AB ( infants or toddlers or young children ) OR AB neonatal OR AB ( adolescents or teenagers or young adults ) OR AB neonate OR AB ( paediatrics or children ) OR AB ( paediatrics or children or child or young person ) OR AB ( boys or girls ) OR AB boyhood OR AB girlhood OR AB youth OR AB ( youths or young people or adolescents or teenagers ) OR AB ( youths or teenagers or juvenile ) OR AB baby OR AB babies OR AB toddlerhood OR AB teens OR AB newborn OR AB puberty OR AB preschool OR AB preschool children OR TI girlhood OR TI youth OR TI ( youths or young people or adolescents or teenagers ) OR TI ( youths or teenagers or juvenile ) OR TI baby OR TI babies OR TI toddlerhood OR TI teens OR TI newborn OR TI puberty OR TI preschool OR TI preschool children OR TI suckling OR AB suckling
