# Supplementary material for: Systematic Review of Childhood Sedentary Behavior Questionnaires: What do We Know and What is Next?
Source: Sports Med. 2016 Aug 31;47(4):677–99. doi: 10.1007/s40279-016-0610-1 (PMC5357243; doi:10.1007/s40279-016-0610-1)
Supplement: Supplementary file 2 — Supplementary material 2 (DOCX 156 kb) [file 40279_2016_610_MOESM2_ESM.docx]

Electronic Supplementary Material Table S1. Description of sedentary behavior questionnaires for youth sorted by age category

A systematic review of childhood sedentary behavior questionnaires: what do we know and what’s next?

Journal: Sports Medicine

Lisan M. Hidding,^1^ Teatske M. Altenburg,^1^ Lidwine B. Mokkink,^2^ Caroline B. Terwee,^2^ Mai J. M. Chin A Paw^1^

^1^Department of Public and Occupational Health, EMGO Institute for Health and Care Research, VU University Medical Center, Amsterdam, the Netherlands

^2^Department of Epidemiology and Biostatistics, EMGO Institute for Health and Care Research, VU University Medical Center, Amsterdam, the Netherlands

E-mail corresponding author: [l.hidding@vumc.nl](mailto:l.hidding@vumc.nl)

Electronic Supplementary Material Table S1. Description of sedentary behavior questionnaires for youth sorted by age category

| Questionnaire | Target population | Construct | | | Format | | | |
| --- | --- | --- | --- | --- | --- | --- | --- | --- |
|  |  | Construct | Setting | Recall period | Dimensions | No. of questions | | Scores |
| **Preschoolers (mean age <6 y)** | | | | | | | | |
| Preschool-aged Children's Physical Activity Questionnaire (Pre-PAQ) (proxy), R, ME [1] | Pre-school children | Time spent in car; watching TV/DVD/video; reading or being read to; playing computer or electronic games | All | Last week; yesterday; last weekend | D | 15-27 questions  (min-max) | Yes/no; hrs./min. | |
| Physical activity and sedentary behavior questionnaire (based on the Canadian Health Measures Survey) (proxy), V [2] | Young children | TV viewing; videos/DVD viewing; computer playing; game console playing; playing handheld devices; stroller time; traveling in motor vehicle | All | Typical weekday; typical weekend day | D | 12 questions | Time spent | |
| KidActive-Q  (proxy), R [3] | Proxy questionnaire for young children | TV watching/playing computer games | All; transportation to and from daycare | Usual; past few months | D + F | ? | Yes/no; frequency and/or duration | |
| Energy Balance Related Behaviors (ERBs) self-administered primary caregivers questionnaire (PCQ), from the ToyBox-study (proxy), R [4] | Pre-school children | Transport; watching TV/DVD/video; playing games on a computer or game console; quiet play | All; leisure time; transport to and from preschool | General weekday; general weekend day; usually | D | 10 questions | 2 different answering formats: 1) time in min.; 2) 10 categories ranging from never to I don’t know (30 minute and 1 hourly intervals) | |
| Physical activity questionnaire for parents (translated from Spanish) (proxy), R, V [5] | Preschool children | Watching TV; playing; lying down in bed or an armchair | All | Usual | D | 1 question | 5 categories ranging from 15 min. to more than 1 hr. | |
| A TV viewing question, R [6] | Children | TV/video viewing | All | Average day | D | 1 question | Hrs./day | |
| Daily Activity Chart, (proxy), V [7] | Children | TV watching | All | Typical week | D + F | 1 question | Child's daily schedule in one hr. intervals | |
| The Direct Estimate (proxy), V [7] | Children | TV watching | Morning; afternoon; evening (6 am-12 noon, 12 noon - 6 pm, 6 pm – midnight) | This time of the year; Monday - Sunday | D | 1 question | Hrs. | |
| **Children (mean age** ≥**6 and <12 y)** | | | | | | | | |
| Questionnaire for measuring length of sleep, television habits and computer habits (proxy and non-proxy), ME [8] | Children in pre-school class (age 6), children in grades 1 (age 7), 4 (age 10), and 8 (age 14), and in the first upper secondary school grade (age 16) | TV habits; computer habits | All | Typical day | D | 2 | Hrs./min./day | |
| Sedentary Behavior and Sleep Scale (SBSS), R, V [9] | Children | Screen time: watching TV, playing video games, using the computer; homework; after school tuition | All | Typical school day; typical weekend day | D | 2 questions | Time spent | |
| TV viewing and organized physical activity questions, R [10] | Children | TV viewing | All | Usual school day; usual weekend day | D | 2 questions | 12 categories ranging from 0 hrs. to 6 or more hrs. (in half-hour segments) | |
| Energy Retention Behavior Scale for Children (ERB-C scale), R [11] | Children | Recreational TV watching; recreational computer or other electronic game behavior | All | General weekday; general weekend day | D | 4 | 5 categories ranging from ≤1hr./day to ≥4hrs./day | |
| Selected physical activity and sedentary behavior items of the Health Behavior School-aged Children (HBSC) questionnaire, R [12] | School-aged children | TV use; computer use; sitting time | Free time; outside school hours | Usual weekday; usual weekend day | D | 6 | 9 categories ranging from none at all to about 7 or more hrs./day, cut of point dichotomization = 2 or more hrs./day | |
| Patient Reported Outcome Measurement Information System (PROMIS), X [13,14] | Children | Playing video or computer games; computer use, watching TV; sitting with friend/family; sitting during transport; texting, talking on the phone; doing homework | All; after school; night; morning; afternoon | Past 7 days; weekday; weekend day; usual weekday; usual weekend day; usual day | D | 21 questions | 5 categories ranging from none to 5 or more hrs. | |
| Canadian Health Measures Survey (CHMS) (proxy), V [15] | Children | Watching TV/videos/playing video games; computer use | All | Usual day | D | 2 questions | Hrs./day | |
| Parent proxy-report of physical activity and sedentary activities (proxy), R, V [16] | Children and adolescents | Watching television/video/DVD; sitting at a computer/playing Nintendo/electronic games; doing homework; playing a musical instrument; reading; playing quietly or performing other quiet activities; traveling by car/public transport | All; transport to school | Typical weekday; typical weekend day; usually | D | 7 questions | 6 categories ranging from never to more than four hrs./day | |
| Self-Administered Physical Activity Checklist (SAPAC), R, V [17,18] | Children | TV/video watching; Computer & games | Before school; after school | Previous day | D | 4 questions | Hrs./min. | |
| HABITS questionnaire, V [19] | Children | TV watching | All | Past month; weekday; weekend day | D | 2 questions | 4 categories ranging from <1hr. to 3 hrs. or more a day | |
| Selected indicators from the  Health Behavior in School-aged Children (HBSC) questionnaire, R, ME [20] | School-aged children | Watching TV/DVDs; computer/console use for playing games; computer use for chatting, internet, emailing, homework; doing homework | Free time | Usual school day; usual weekend day | D | 8 questions | 9 categories ranging from none to approx. 7 hrs. or more | |
| Questions from the National Longitudinal Survey of Children and Youth (proxy), V [21] | Children and youth | Watching TV;  using a computer or playing video games | All | ? | D | 2 questions | 3 categories ranging from one hr. or less to more than three hrs. | |
| The Adolescents Sedentary Activities Questionnaire (ASAQ), R [22] | Adolescents | Watching television/videos/DVD; computer use; e-games and e-communication; studying; reading; sitting with friends; telephone use; listening or playing music; motorized travel; hobbies and crafts | Before school; after school | Usual week | D | 79 questions | Hrs./min. | |
| The Eating and Activity Questionnaire Trial (Project EAST), R, ME, V [23] | Middle school children | Watching television/videos; playing video games; computer use including time spent on the internet and playing games | Outside school hours | Usual | D | 5 questions | Time spent | |
| A modified question of the Youth Risk Behavior Survey (YRBS), R, V [23] | Middle school children | Watching television | All | Usual school day | D | 1 question | Time spent | |
| TV viewing items of the Health Behavior in School-Aged Children survey (HBSC), R, V [24] | School-aged children | TV viewing (including videos) | Free time; all | Usual weekday; usual weekend day | D | 2 questions | 9 categories ranging from none at al to about 7 or more hrs. a day | |
| Children's Leisure Activities Study Survey Chinese-version questionnaire (CLASS-C), R, V [25] | Children | Sedentary activities | Leisure time; school PE classes; transportation-related activities | Last week; weekday; weekend day | D + F | 14 sedentary items | Time spent | |
| Quantification de l'Activite Physique en Altitude Chez le Enfants (QAPACE), R [26] | Children | Classroom; studying or doing homework | School; at home | Representative school week | D | 2 questions | 2 different answering formats: 1) 4 categories ranging from 5 hrs. to 8+ hrs.; 2) 7 categories, ranging from 15 min. to 120+ min. | |
| Sedentary behavior items from a new questionnaire to identify usual patterns of physical activity, R, V [27] | Children | Sitting; TV/VCR/video games | All | Typical school day; typical weekend day | D + F | Three 24 hr. timetables | Hrs./day | |
| Sedentary behavior items from a new questionnaire to identify usual patterns of physical activity (proxy), R, V [27] | Children | Sitting; TV/VCR/video games | All | Typical school day; typical weekend day | D + F | 4 questions | Hrs./day | |
| The ENERGY-project Dietary and Physical Activity Habits of Children -child questionnaire, R, ME, V [28] | Children | Transport; TV watching; computer use; activities during break | Free time:  to school; at school | Usual weekday; usual weekend day; yesterday; today | D + F | 10 questions | 4 different answering formats: 1) 6 categories ranging from never to 5 days per week; 2) by bike, by foot, by car, by public transport; 3) I usually spend the time sitting, I usually spend the time walking and moving around, I usually spend the time doing sports or similar activities; 4) 9 categories ranging from none at al to 4,0 or more hrs./day | |
| Youth Activity Profile (YAP), V [29] | Youth | Sedentary habits; TV time; playing videogames; using the computer; using a cell phone; overall sedentary behavior | Out-of-school; at home | Past 7 days | D + F | 5 questions | 2 different answering formats: 1) 5 categories ranging from I didn't … at all to more than 3 hrs./day; 2) 5 categories, ranging from I spend almost none of my free time sitting to I spend almost all of my free time sitting | |
| Girls health Enrichment Multi-site Studies (GEMS) Activity Questionnaire (GAQ), R [30,31] | African American girls | Watching TV or video; computer or video games; arts and crafts; board games; homework or reading; talking on phone or hanging out; listening to music or playing an instrument | All | Yesterday; usually | D | 14-28 questions (min-max) | 5 categories ranging from none to more than 3 hrs. | |
| UP4FUN child questionnaire, R, ME [32] | Children | TV/DVD; computer/game console; breaking up TV/DVD; breaking up computer/game console;  breaking up sitting at school | All; leisure activities; at school | Yesterday; usual weekend day; usual weekday; usually | D + F | 5 questions | 2 different answering formats: 1) 10 categories ranging from none to 4 hrs./day or more; 2)  5 categories ranging from never to more than 4 times/hr. sitting | |
| **Older children and adolescents (mean age** ≥**12 y)** | | | | | | | | |
| Measures of out-of-school sedentary and travel behaviors of the international Healthy Environments and active living in teenagers – Hong kong (iHealt(H)) study, R, ME [33] | Adolescents | Watching TV/videos/DVDs; playing sedentary computer or video games; using internet, emailing or other electronic media for leisure; doing homework; reading a book or a magazine not for school; and riding a car, bus or other vehicle; transportation behavior | Out-of-school | Usual school day; average school week | D + F | 14 questions | 2 different answering formats: 1) 7 categories ranging from 0 hrs. to 4 or more hrs./day; 2) 6 categories ranging from 0 to 5 days | |
| School Health Action Planning and Evaluation System (SHAPES) physical activity questionnaire, R [34] | Students | Watching TV/movies; playing video/computer games; surfing the internet; talking on the phone | All | 7 day recall | D | ? | Hrs./wk. | |
| Newly developed questionnaire on total sedentary time, R, V [35] | Adolescents | TV watching; gaming; using the computer; reading; hobbies; socializing; listening to music; during meals; motorized transport; school work at home; telephone use | Free time; to and from school; at school; during lessons; all | Past 7 days | D | 25 questions | 3 different answering formats: 1) 11 categories ranging from none to more than 7 hrs./day; 2) 14 categories ranging from none to more than 7 hrs./day; 3) min./day | |
| Adolescent Sedentary Activity Questionnaire (ASAQ) (Brazilian version), R, ME [36] | Adolescents | Screen recreation: TV, DVDs/videos, computer use leisure, go to the movies;  Educational: computer use homework, homework study without computer, take a course or private lessons, classroom sitting; Cultural: reading for leisure, handicraft or other manual hobby, play/practice a musical instrument;  Social: chat with friends/mess around/relax, stay on the telephone, listen to music, have class, go to church; Transport: travel or move | All | Typical weekend; typical week | D | 91 questions | Hrs./min. | |
| Flemish Physical Activity Computer Questionnaire (FPACQ), R, ME [37] | Children | Watching TV/video; playing video games | All | Usual weekday; usual weekend day | D | 2 questions | Hrs./day | |
| The International Physical Activity Questionnaire (IPAQ) self-administered short version, R [38] | Young and middle aged adults (15-69) | Sitting | All | Last week (weekdays) | D + F | 1 question | Hrs./min./day (Amount of time for at least 10 min.) | |
| International Physical Activity Questionnaire - Short Form (IPAQ-SF) (Chinese version), R, V [39] | Adults | Sitting | All | Last 7-days | D | 1 question | Hrs./min./day, don't know/not sure | |
| A questionnaire to measure a broad range of sedentary activities, V [40] | Adolescents | Watching TV/Video, or playing video games; using computer for fun or study; doing homework/study (not on computer) or reading for fun; talking on the telephone, sitting with friends or hanging out; doing hobbies or crafts or music lessons/practice; traveling in a car, bus, ferry or train; going to the cinema | Before and after school | Usual weekday; usual weekend days | D + F | 13 questions | Min./day | |
| 1999 Youth Risk Behavior Survey (YRBS) questionnaire, R [41] | Youth | TV viewing | All | Usual school day | D | 1 question | ? | |
| Korean Youth Risk Behavior (KYRBS) survey, X [42] | Youth | Watching TV; using a computer for internet games, etc. | All | Last 7 days | D | 2 questions | Time spent | |
| Child and Adolescent Physical Activity and Nutrition survey (CAPANS-PA) recall questionnaire, R [43] | Children and adolescents | Watch TV/videos/DVDs; play video games; use computer for fun; study or homework; read for fun; chat on phone; hanging out; hobbies/crafts; music lessons/practice; travel in car, train, bus, boat/ferry; go to church; Saturday school; out-of-school tutoring | After finishing school | Last 7 days | D | 42 questions | Hrs./min. | |
| Healthy Lifestyle Europe by Nutrition in Adolescence (HELENA) screen time-based sedentary behavior questionnaire, R, V [44] | Adolescents | TV viewing; computer games; console (video) games; internet for non-study reasons (hobbies); internet for study reasons; study time | All | Habitual /usual weekday; habitual /usual weekend day | D | 12 questions | 7 categories ranging from nothing to more than 4 hrs. | |
| Modified 3-day Self-Administered Physical Activity Checklist (SAPAC), V [45] | Children | TV/video; video games and computer games; computer and internet use; talking on the phone | Out-of-school hours; morning; between lunch and dinner; after dinner | 3-day recall | D | 20 questions | Hrs./min. | |
| Activity Questionnaire for Adults and Adolescents (AQuAA), R, V [46] | Adults and adolescents | Traveling by car/motor scooter/public transport; sedentary leisure time activities | Leisure time; commuting to and from school/work | Past 7 days | D + F | 2-10 questions (min-max) | 2 different answering formats: 1) no. days; 2) hrs./min. | |

R = Reliability data available; ME = Measurement error data available; V = Construct validity data available; X = No construct reliability, measurement error, or construct validity data available; D = Duration; F = Frequency; ? = Indicating not reported

References

1. Dwyer GM, Hardy LL, Peat JK, et al. The validity and reliability of a home environment preschool-age physical activity questionnaire (Pre-PAQ). Int J Behav Nutr Phys Act. 2011;8:86.

2. Sarker H, Anderson LN, Borkhoff CM, et al. Validation of parent-reported physical and sedentary activity by accelerometry in young children. BMC Res Notes. 2015;8:735.

3. Bonn SE, Surkan PJ, Trolle Lagerros Y, et al. Feasibility of a novel web-based physical activity questionnaire for young children. Pediatr Rep. 2012;4:127–9.

4. González-Gil EM, Mouratidou T, Cardon G, et al. Reliability of primary caregivers reports on lifestyle behaviours of European pre-school children: The ToyBox-study. Obes Rev. 2014;15:61–6.

5. Bacardi-Gascón M, Reveles-Rojas C, Woodward-Lopez G, et al. Assessing the validity of a physical activity questionnaire developed for parents of preschool children in Mexico. J Health Popul Nutr. 2012;30:439–46.

6. Janz KF, Broffitt B, Levy SM. Validation evidence for the Netherlands physical activity questionnaire for young children: the Iowa Bone Development Study. Res Q Exerc Sport. 2005;76:363–9.

7. Anderson DR, Field DE, Collins PA, et al. Estimates of young children’s time with television: a methodological comparison of parent reports with time-lapse video home observation. Child Dev. 1985;56:1345–57.

8. Garmy P, Jakobsson U, Nyberg P. Development and psychometric evaluation of a new instrument for measuring sleep length and television and computer habits of Swedish school-age children. J Sch Nurs. 2012;28:138–43.

9. Mellecker RR, McManus AM, Matsuzaka A. Validity and reliability of the sedentary behavior and sleep scale (SBSS) in young Hong Kong Chinese children. Asian Journal of Exercise & Sports Science. 2012;9:21–36.

10. Salmon J, Campbell KJ, Crawford DA. Television viewing habits associated with obesity risk factors: a survey of Melbourne schoolchildren. Med J Aust. 2006;184:64–7.

11. Chen S-W, Cheng C-P, Wang R-H, et al. Development and psychometric testing of an energy retention behavior scale for children. J Nurs Res. 2015;23:47–55.

12. Bobakova D, Hamrik Z, Badura P, et al. Test–retest reliability of selected physical activity and sedentary behaviour HBSC items in the Czech Republic, Slovakia and Poland. Int J Public Health. 2014;60:59–67.

13. Tucker CA, Bevans KB, Teneralli RE, et al. Self-reported pediatric measures of physical activity, sedentary behavior, and strength impact for PROMIS: conceptual framework. Pediatr Phys Ther. 2014;26:376–84.

14. Tucker CA, Bevans KB, Teneralli RE, et al. Self-reported pediatric measures of physical activity, sedentary behavior, and strength impact for PROMIS: item development. Pediatr Phys Ther. 2014;26:385–92.

15. Colley RC, Wong SL, Garriguet D, et al. Physical activity, sedentary behaviour and sleep in canadian children: parent-report versus direct measures and relative associations with health risk. Health Rep. 2012;23:45–52.

16. Bringolf-Isler B, Mäder U, Ruch N, et al. Measuring and validating physical activity and sedentary behavior comparing a parental questionnaire to accelerometer data and diaries. Pediatr Exerc Sci. 2012;24:229–45.

17. Brown TD, Holland BV. Test-retest reliability of the self-assessed physical activity checklist. Percept Mot Skills. 2004;99:1099–102.

18. Sallis JF, Strikmiller PK, Harsha DW, et al. Validation of interviewer- and self-administered physical activity checklists for fifth grade students. Med Sci Sports Exerc. 1996;28:840–51.

19. Wright ND, Groisman-Perelstein AE, Wylie-Rosett J, et al. A lifestyle assessment and intervention tool for pediatric weight management: the HABITS questionnaire. J Hum Nutr Diet. 2011;24:96–100.

20. Liu Y, Wang M, Tynjälä J, et al. Test-retest reliability of selected items of Health Behaviour in School-aged Children (HBSC) survey questionnaire in Beijing, China. BMC Med Res Methodol. 2010;10:73.

21. Sithole F, Veugelers PJ. Parent and child reports of children’s activity. Health Rep. 2008;19:19–24.

22. Hardy LL, Booth ML, Okely AD. The reliability of the Adolescent Sedentary Activity Questionnaire (ASAQ). Prev Med. 2007;45:71–4.

23. Schmitz KH, Harnack L, Fulton JE, et al. Reliability and validity of a brief questionnaire to assess television viewing and computer use by middle school children. J Sch Health. 2004;74:370–7.

24. Vereecken CA, Todd J, Roberts C, et al. Television viewing behaviour and associations with food habits in different countries. Public Health Nutr. 2006;9:244–50.

25. Huang YJ, Wong SHS, Salmon J. Reliability and validity of the modified Chinese version of the Children’s Leisure Activities Study Survey (CLASS) questionnaire in assessing physical activity among Hong Kong children. Pediatr Exerc Sci. 2009;21:339–53.

26. Barbosa N, Sanchez CE, Vera JA, et al. A physical activity questionnaire: reproducibility and validity. J Sports Sci Med. 2007;6:505–18.

27. Ching PLYH, Dietz WH. Reliability and validity of activity measures in preadolescent girls. Pediatr Exerc Sci. 1995;7:389–99.

28. Singh AS, Vik FN, Chinapaw MJM, et al. Test-retest reliability and construct validity of the ENERGY-child questionnaire on energy balance-related behaviours and their potential determinants: the ENERGY-project. Int J Behav Nutr Phys Act. 2011;8:136.

29. Saint-Maurice PF, Welk GJ. Validity and calibration of the youth activity profile. PLoS One. 2015;10:e0143949.

30. Treuth MS, Sherwood NE, Butte NF, et al. Validity and reliability of activity measures in African-American girls for GEMS. Med Sci Sports Exerc. 2003;35:532–9.

31. Treuth MS, Sherwood NE, Baranowski T, et al. Physical activity self-report and accelerometry measures from the Girls health Enrichment Multi-site Studies. Prev Med. 2004;38:43–9.

32. Vik FN, Lien N, Berntsen S, et al. Evaluation of the UP4FUN intervention: a cluster randomized trial to reduce and break up sitting time in European 10-12-year-old children. PLoS One. 2015;10:e0122612.

33. Cerin E, Sit CHP, Huang Y-J, et al. Repeatability of self-report measures of physical activity, sedentary and travel behaviour in Hong Kong adolescents for the iHealt(H) and IPEN - adolescent studies. BMC Pediatr. 2014;14:142.

34. Wong SL, Leatherdale ST, Manske SR. Reliability and validity of a school-based physical activity questionnaire. Med Sci Sports Exerc. 2006;38:1593–600.

35. Busschaert C, De Bourdeaudhuij I, Van Holle V, et al. Reliability and validity of three questionnaires measuring context-specific sedentary behaviour and associated correlates in adolescents, adults and older adults. Int J Behav Nutr Phys Act. 2015;12:117.

36. de Fátima Guimarães R, da Silva MP, Legnani E, et al. Reproducibility of adolescent sedentary activity questionnaire (ASAQ) in Brazilian adolescents. Brazilian Journal of Kinanthropometry and Human Performance. 2013;15:276–85.

37. Philippaerts RM, Matton L, Wijndaele K, et al. Validity of a physical activity computer questionnaire in 12- to 18-year-old boys and girls. Int J Sports Med. 2006;27:131–6.

38. Rangul V, Holmen TL, Kurtze N, et al. Reliability and validity of two frequently used self-administered physical activity questionnaires in adolescents. BMC Med Res Methodol. 2008;8:47.

39. Wang C, Chen P, Zhuang J. Validity and reliability of International Physical Activity Questionnaire-Short Form in Chinese youth. Res Q Exerc Sport. 2013;84:S80–6.

40. Hardy LL, Bass SL, Booth ML. Changes in sedentary behavior among adolescent girls: a 2.5-year prospective cohort study. J Adolesc Health. 2007;40:158–65.

41. Brener ND, Kann L, McManus T, et al. Reliability of the 1999 youth risk behavior survey questionnaire. J Adolesc Health. 2002;31:336–42.

42. Park JM, Han AK, Cho YH. Construct equivalence and latent means analysis of health behaviors between male and female middle school students. Asian Nurs Res. 2011;5:216–21.

43. Strugnell C, Renzaho A, Ridley K, et al. Reliability of the modified child and adolescent physical activity and nutrition survey, physical activity (CAPANS-PA) questionnaire among Chinese-Australian youth. BMC Med Res Methodol. 2011;11:122.

44. Rey-López JP, Ruiz JR, Ortega FB, et al. Reliability and validity of a screen time-based sedentary behaviour questionnaire for adolescents: The HELENA study. Eur J Public Health. 2012;22:373–7.

45. Affuso O, Stevens J, Catellier D, et al. Validity of self-reported leisure-time sedentary behavior in adolescents. J Negat Results Biomed. 2011;10:2.

46. Chinapaw MJM, Slootmaker SM, Schuit AJ, et al. Reliability and validity of the Activity Questionnaire for Adults and Adolescents (AQuAA). BMC Med Res Methodol. 2009;9:58.
